# Clustering and machine learning-based integration identify cancer associated fibroblasts genes’ signature in head and neck squamous cell carcinoma

**DOI:** 10.3389/fgene.2023.1111816

**Published:** 2023-03-30

**Authors:** Qiwei Wang, Yinan Zhao, Fang Wang, Guolin Tan

**Affiliations:** ^1^ Department of Otolaryngology Head and Neck Surgery, Third Xiangya Hospital, Central South University, Changsha, Hunan, China; ^2^ Xiangya School of Nursing, Central South University, Changsha, Hunan, China; ^3^ Department of Otorhinolaryngology/Head and Neck Surgery, University Hospital Rechts der Isar, Technical University of Munich, Munich, Bavaria, Germany; ^4^ Third Xiangya Hospital, Central South University, Changsha, China

**Keywords:** HNSCC, cancer-associated fibroblasts (CAFs), machine learning, the tumor microenvironment, prognosis

## Abstract

**Background:** A hallmark signature of the tumor microenvironment in head and neck squamous cell carcinoma (HNSCC) is abundantly infiltration of cancer-associated fibroblasts (CAFs), which facilitate HNSCC progression. However, some clinical trials showed targeted CAFs ended in failure, even accelerated cancer progression. Therefore, comprehensive exploration of CAFs should solve the shortcoming and facilitate the CAFs targeted therapies for HNSCC.

**Methods:** In this study, we identified two CAFs gene expression patterns and performed the single‐sample gene set enrichment analysis (ssGSEA) to quantify the expression and construct score system. We used multi-methods to reveal the potential mechanisms of CAFs carcinogenesis progression. Finally, we integrated 10 machine learning algorithms and 107 algorithm combinations to construct most accurate and stable risk model. The machine learning algorithms contained random survival forest (RSF), elastic network (Enet), Lasso, Ridge, stepwise Cox, CoxBoost, partial least squares regression for Cox (plsRcox), supervised principal components (SuperPC), generalised boosted regression modelling (GBM), and survival support vector machine (survival-SVM).

**Results:** There are two clusters present with distinct CAFs genes pattern. Compared to the low CafS group, the high CafS group was associated with significant immunosuppression, poor prognosis, and increased prospect of HPV negative. Patients with high CafS also underwent the abundant enrichment of carcinogenic signaling pathways such as angiogenesis, epithelial mesenchymal transition, and coagulation. The MDK and NAMPT ligand–receptor cellular crosstalk between the cancer associated fibroblasts and other cell clusters may mechanistically cause immune escape. Moreover, the random survival forest prognostic model that was developed from 107 machine learning algorithm combinations could most accurately classify HNSCC patients.

**Conclusion:** We revealed that CAFs would cause the activation of some carcinogenesis pathways such as angiogenesis, epithelial mesenchymal transition, and coagulation and revealed unique possibilities to target glycolysis pathways to enhance CAFs targeted therapy. We developed an unprecedentedly stable and powerful risk score for assessing the prognosis. Our study contributes to the understanding of the CAFs microenvironment complexity in patients with head and neck squamous cell carcinoma and serves as a basis for future in-depth CAFs gene clinical exploration.

## Introduction

Head and Neck Squamous Cell Carcinoma (HNSCC) is an aggressive tumor associated with poor prognosis. There are more than 600,000 new cases are diagnosed worldwide each year (Bray et al., 2018). A hallmark signature of the tumor microenvironment (TME) in HNSCC is abundantly infiltration of cancer-associated fibroblasts (CAFs), which facilitate HNSCC progression (Custódio et al., 2020). CAFs could secrete exosomes which assist cell to-cell communication with TME thereby remodeling extracellular matrix (ECM) (Custódio et al., 2020). Biologically, the characteristics of cell stromal appears to be no difference between HNSCC patients, suggesting there is likely to exist a common weakness in stromal compartment which could be potential CAFs treatment targets (Puram et al., 2017).

Previous studies revealed an immunosuppressive role of CAFs, which could strongly induce the dysfunction of T cells and macrophages (Thomas and Massagué, 2005; Tauriello et al., 2018). The reason is attributable to CAFs secrete ECM components hence developing a dense fibrotic barrier in the tumor (Drifka et al., 2016). Benefit from bulk and single-cell RNA sequencing, A lots of new CAFs biomarkers have been figured out. Targeted therapy for CAFs also has made a breakthrough in hepatocellular carcinoma (Yin et al., 2019). However, some targeted CAFs clinical trials ended in failure, even accelerated cancers progression (Catenacci et al., 2015; Van Cutsem et al., 2020). Therefore, more comprehensive exploration of CAFs should solve the shortcoming and facilitate the targeted therapies in HNSCC.

Here, we collect 868 HNSCC samples from multi-dimensional common datasets, using clustering and machine learning method to detect the correlation between CAFs biological functions and clinical characteristics in HNSCC. We hope to find some specific molecular mechanisms to understand tumor progression and improve clinical management in head and neck squamous cell carcinoma.

## Methods

### HNSCC dataset source and processing

We summarized 31 CAFs genes from Kürten, C. H. L. et al. (Kürten et al., 2021) single-cell RNA sequencing research and Chakravarthy, A.et al. (Chakravarthy et al., 2018) bulk-RNA sequencing research. Total 868 samples from The Cancer Genome Atlas HNSCC (TCGA-HNSC) cohort and Gene Expression Omnibus cohort (GSE65858, GSE41613) were involved in our study. We constructed a Combined cohort by filtering common genes from GSE41613, GSE65858, and TCGA cohorts. We used “combat” R software package to remove the batch effects. The results were validated using internal and external cohort.

### Construction of molecular types and score system based on the CAFs genes

We used R package “ClassDiscovery” to distinguish CAFs genes’ expression pattern in the Combined cohort. The single‐sample gene set enrichment analysis (ssGSEA) method was used to construct CAFs related score system CafS.

### Estimation of immune infiltration

We used ssGSEA method and CIBERSORT algorithm (Newman et al., 2015) to evaluated absolute abundance of multiple immune cell populations. R package “ESTIMATE” was performed to calculated stromal score.

### Single-cell analysis

We downloaded GSE164690 single cell cohort from Gene Expression Omnibus database. R package “Seurat” (Butler et al., 2018) was used to analysis single cell database. We filtered mitochondrial genes with parameter <10%. We selected highly variable genes with parameter nfeatures = 2000. These variable genes were used as inputs for PCA. Dims = 1:15 was used to FindNeighbors and resolution = 0.5 were used for FindClusters. We identified 18 primary clusters, and cluster analysis were performed by the RunUMAP function. We found differentially expressed genes (DEGs) for each cluster with parameters min.pct = 0.25 & thresh.use = 0.25. We compared DEGs and annotated CAFs (FAP, MMP11, PDGFRA, PDGFRB, ADAMTS2, SFPR2); Endothelial cell (PLVAP, KDR, PTPRB) in clusters. “Single R” package was used to annotate remaining clusters. MuSic deconvolution method (Wang et al., 2019) was used to calculate the proportion of CAFs. The CellChat method (Jin et al., 2021) was used to analysis cellular communication.

### Construction and verification of the prognostic model

LASSO algorithm was first used to filter the candidate prognostic CAFs genes. We then integrated 10 machine learning algorithms and 107 algorithm combinations to construct most accurate and stable risk model. The machine learning algorithms contained random survival forest (RSF), elastic network (Enet), Lasso, Ridge, stepwise Cox, CoxBoost, partial least squares regression for Cox (plsRcox), supervised principal components (SuperPC), generalised boosted regression modelling (GBM), and survival support vector machine (survival-SVM). All models were detected in four datasets (GSE41613, GSE65858, TCGA-HNSC, and Combined cohort). We calculated the concordance index (C-index) across all datasets, and the model with the highest average C-index was considered optimal. We used the optimal average C-index model machine learning algorithm to validate the robustness of prognostic model in the external cohort GSE42743.

### Cell lines and quantitative real-time PCR assay

HNSCC cell lines CAL-27, FaDu and normal nasopharyngeal epithelial cell line (NP69) were obtained from National Collection of Authenticated Cell Cultures. For reverse transcription, 2 μg of total RNA was used to synthesize cDNA with a cDNA Synthesis Kit. β-actin was used as an internal control. The PGAM1 forward sequence of primer was 5-AAA​CGC​AGG​ACA​GTC​TGA​TGC-3, and reverse sequence of primer was 5-CCG​TCT​GCA​GCT​ACA​ACT​CA-3. The ENO1 forward sequence of primer was 5-CGA​GAC​CCA​GTG​GCT​AGA​AGT​T-3, and reverse sequence of primer was 5-AAG​TGC​CAC​CCA​GAG​AGG​AC-3. The β-actin forward sequence of primer was 5-CAT​TAA​GGA​GAA​GCT​GTG​CT-3, and reverse sequence of primer was 5-GTT​GAA​GGT​AGT​TTC​GTG​GA-3.

### Statistical analysis

All statistical analysis and bioinformatics methods used R (V4.1.2, https://www.r-project.org/) or GraphPad Prism 9.4 software. The correlation analysis was conducted using Pearson method. The Wilcoxon test were performed to compare continuous variables and ordered categorical variables.

### Data and code availability statements

All datasets used in this study are available in public database. The codes supporting the conclusions of this article could provide by reasonable request to corresponding author.

## Result

### Workflow of this study

**FIGURE 1:**
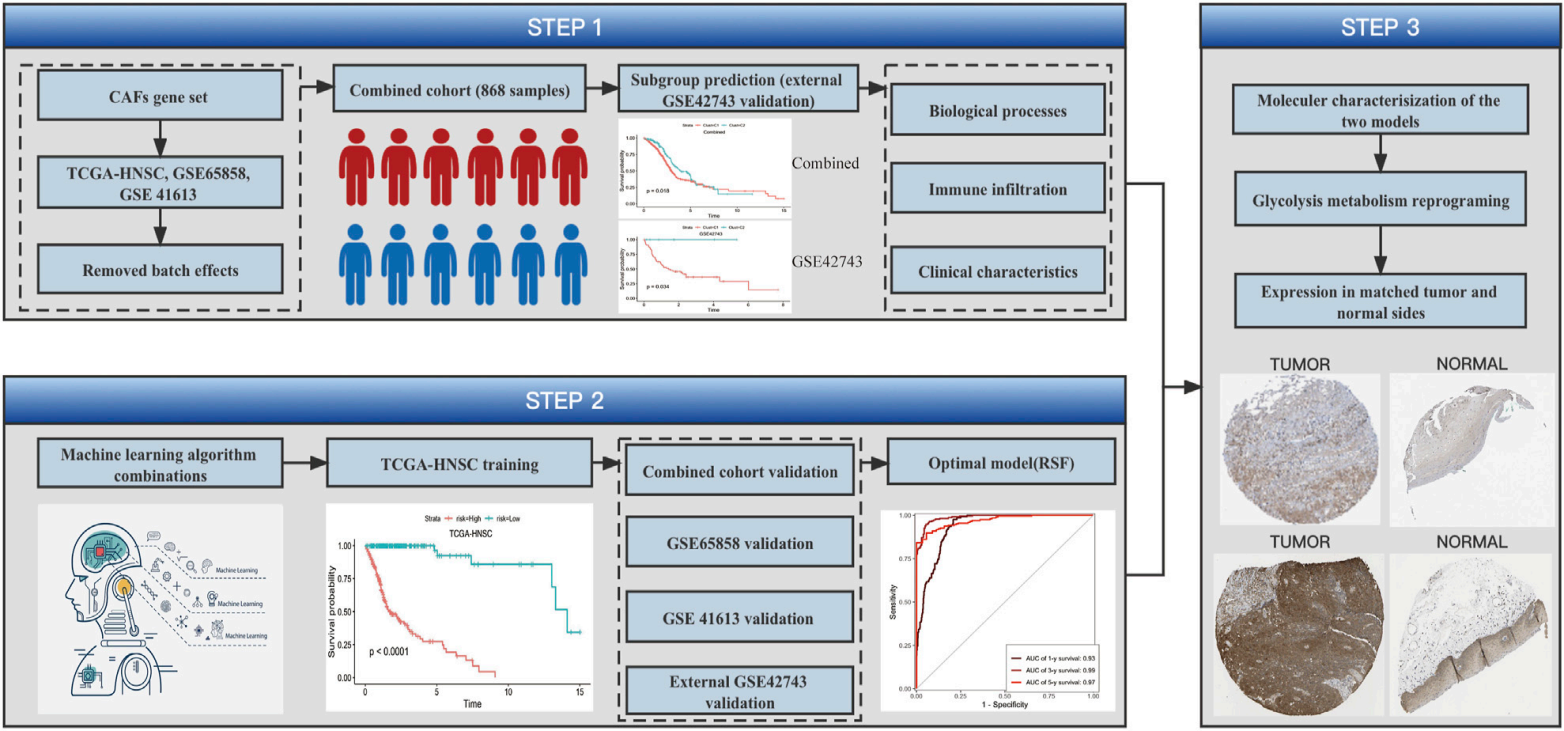
Workflow. The workflow of HNSCC cancer-associated fibroblasts signature analysis.

The study of HNSCC cancer-associated fibroblasts signature analysis is listed in Figure 1 workflow.

### Enrichment analysis in 31 CAFs genes

**FIGURE 2:**
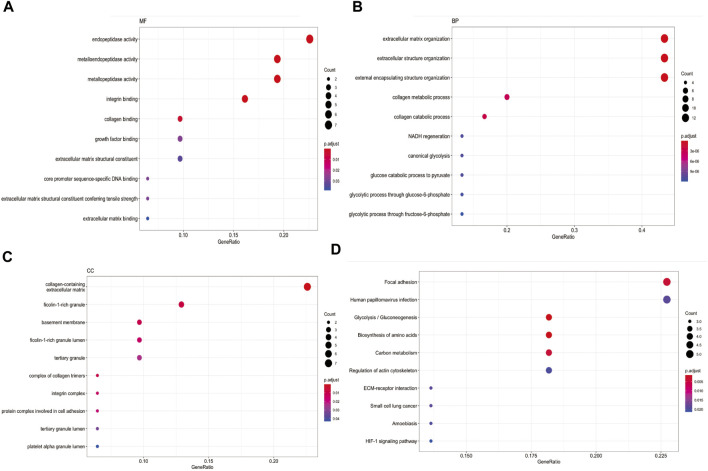
Enrichment analysis in 31 CAFs genes **(A)** MF function analysis for 31 CAFs genes. **(B)** BP function analysis for 31 CAFs genes. **(C)** CC function analysis for 31 CAFs genes. **(D)** KEGG pathway analysis for 31 CAFs genes.

We collected 31 CAFs genes from Kürten, C. H. L. et al. (Kürten et al., 2021) single-cell RNA sequencing research and Chakravarthy, A.et al. (Chakravarthy et al., 2018) RNA sequencing research. They are GAPDH, ENO1, ITGA6, PGK1, TGFBI, ACTN1, FTH1, KDELR2, CD82, SSR3, A2M, PHLDA1, TSC22D1, ISG15, PRSS23, PGAM1, SFRP2, PDGFRB, CEBPB, TNFRSF12A, MMP9, SNA12, ADAMTS2, MMP11, MMP12, COL4A6, STEAP1, ITGAX, ADAMTS14, TLL1 and COL4A4. Some of them have long been recognized as CAFs biomarker. For example, CAFs marker matrix metalloproteinase 11 (MMP11) can be delivered into gastric cancer cells to promote migration (Xu et al., 2019). In addition, CAFs could express MMP9 to enhance proangiogenic phenotype thereby facilitating cancer cell invasion ability in HNSCC (Li et al., 2022). We used “clusterProfiler” R package (Yu et al., 2012) to plot enrichment landscape(Figures 2A–D). Gene Ontology (GO) analysis revealed 31 CAFs genes were mainly enriched in functions such as endopeptidase activity, extracellular matrix organization and collagen-containing extracellular matrix. Kyoto Encyclopedia of Genes and Genomes (KEGG) analysis revealed 31 CAFs genes functions were mainly involved in pathway of focal adhesion. These enrichment function results indicated CAFs genes could play a cellular barrier role in medicine effects by regulating extracellular matrix (Lin et al., 2022).

### Clustering analysis identified two CAFs patterns

**FIGURE 3:**
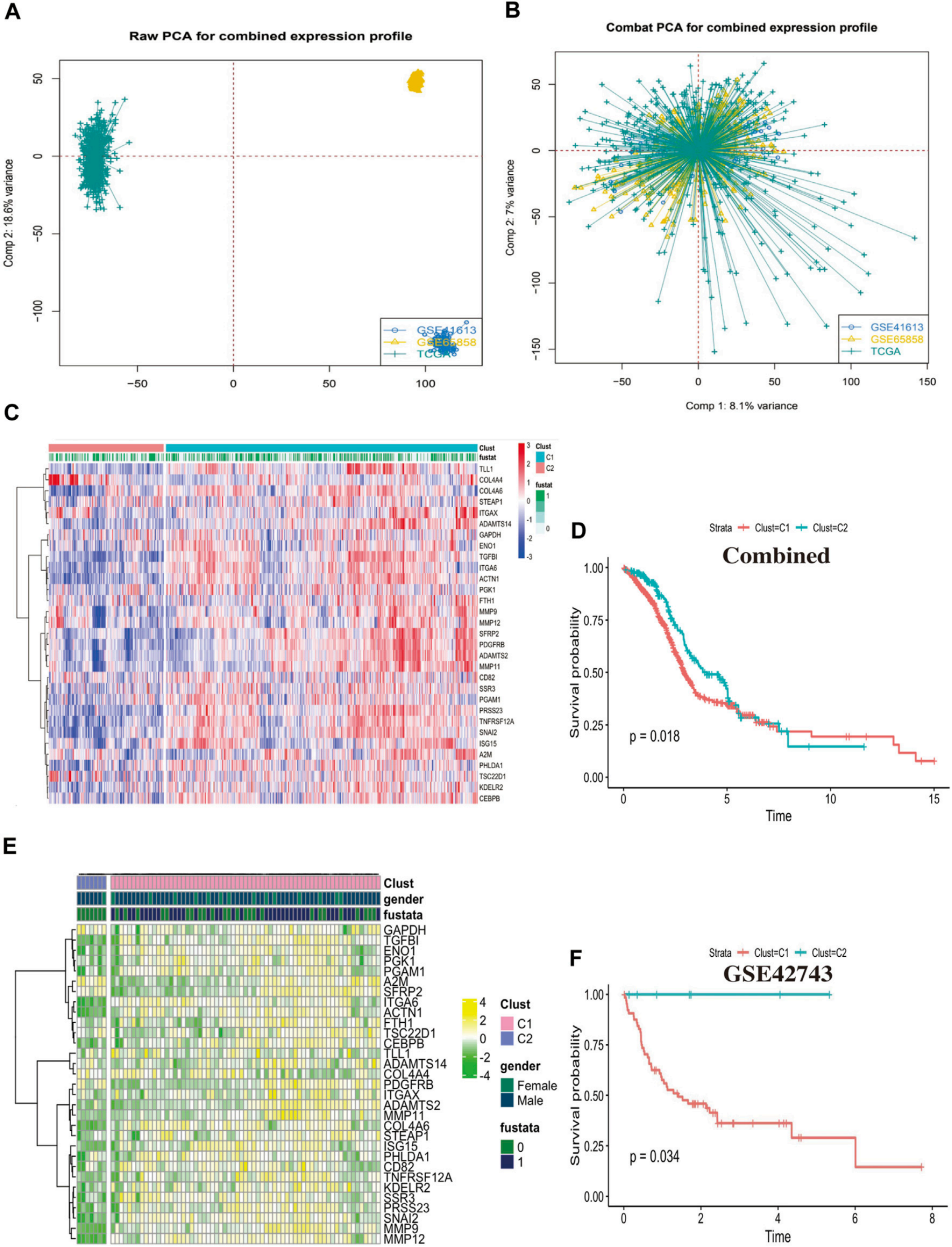
Clustering analysis identified two CAFs patterns. **(A)** Principal component analysis (PCA) showed the gene expression profile in the combined cohort, before elimination of the batch effects. **(B)** Principal component analysis (PCA) showed the gene expression profile in the combined cohort after elimination of the batch effects. **(C)** The heatmap displays the differential expression between the two groups of the 31 cancer associated fibroblasts (CAFs) genes, C1 cluster, C2 cluster, “1” means dead, “0” means alive, “fustat” means survival status. **(D)** The Kaplan-Meier plot displays significant differences survival rate among the two kinds of CAFs phenotypes in the Combined cohorts. C1 was worse than C2 (log rank *p* = 0.018), unit of Time (years). **(E)** The heatmap displays the differential expression between the two groups of the 31 cancer associated fibroblasts (CAFs) genes in the external cohort GSE42743. **(F)** The Kaplan-Meier plot displays the same trend of significant differences survival rate among the two kinds of CAFs phenotypes in the external cohort GSE42743 (log rank *p* = 0.034).

We collected a total of 868 head and neck squamous cell carcinoma samples from TCGA and GEO cohorts to conjoint analysis, the tabular format of clinical sample information covered by this study, which are presented in Supplementary Figures S1A–C. We used “combat” software package to avoid the batch effects, the gene expression profile of each cohort is dispersive (Figure 3A), after elimination of the batch effects, the profile was agminated (Figure 3B). We identified two different CAFs patterns using R package “ClassDiscovery” and labeled as C1 and C2. We plotted a heat map which showed 31 CAFs genes was differential expression in Clust-C1 and Clust-C2 (Figure 3C). Then, after removing unreliable and incomplete clinical data, we analyzed survival prognosis between these two subtypes. The C1 cluster presented particularly survival disadvantage, conversely, the C2 cluster showed exceedingly survival benefit (log-rank, *p* = 0.018; Figure 3D). Similarly, this modification pattern also was observed in the external cohort of GSE42743, clustering analysis identified two similar CAFs related subtypes reminiscent of those observed in the previous combined cohort (Figure 3E). Clust_C1 also exhibits shorter survival than Clust_C2 (log rank *p* = 0.034, Figure 3F). These results suggested there might exist two CAFs related subtypes which could classify HNSCC patients’ survival time.

### Construct a score system CafS to evaluate 31 CAFs genes expression and classify HNSCC clinical characteristics

**FIGURE 4:**
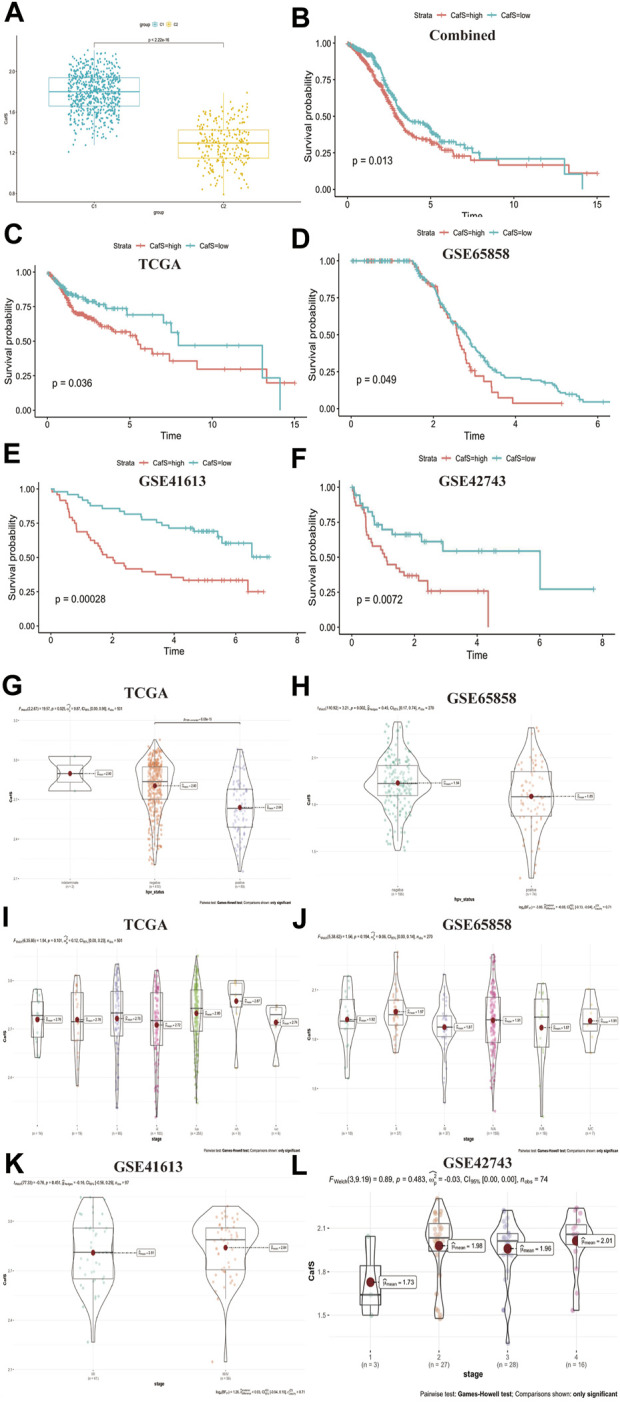
Construct a score system named CafS to evaluate 31 CAFs genes expression and classify HNSCC clinical characteristics **(A)** CafS in the groups of C1 and C2; combined database; *p* < 2.2e-16. **(B–F)** The Kaplan-Meier plot displays significant differences of survival time among the high-CafS and low-CafS groups in the Combined, TCGA, GSE65858, GSE41613, and GSE42743 cohort, respectively. High group was worse than low group, log rank *p* = 0.013, 0.036, 0.049, 0.00028, 0.0072. **(G)** CafS in TCGA cohort among the group of HPV positive and HPV negative, *p* = 6e-10. **(H)** CafS in GSE65858 cohort among the group of HPV positive and HPV negative, *p* = 0.002. **(I–L)** CafS in TCGA, GSE65858, GSE41613, and external cohort GSE42743; among the group of stages; respectively, (*p* = 0.101, 0.194, 0.451, 0.483).

To further explored 31 CAFs genes expression functions in head and neck squamous cell carcinoma, we used the single‐sample gene set enrichment analysis (ssGSEA) method to construct a score system CafS which represented the quantification of these 31 CAFs genes. We found CafS in C1 cluster was significantly higher than C2 (*t*-test, *p* < 2.22e-16; Figure 4A). According the CafS, we divided survival cohort samples into high and low group by the optimal cut-off value, we found CafS was a prognostic factor (log-rank, *p* = 0.013; Figure 4B). In the internal TCGA, GSE41613 and GSE65858 cohorts, high CafS indicated worse survival (log-rank, *p* = 0.036; *p* = 0.00028; *p* = 0.049; respectively, Figures 4C–E). In the external cohort, high CafS also predicted bad outcoming (Figure 4F). These results proved poor prognosis for patients with high CafS. Ang, K. K. et al. (Ang et al., 2010) found there was significant survival advantages in HNSCC patients with HPV (+) comparing to HPV (-). In our study, we found that the CafS level in the HPV (-) group was significantly higher than that in the HPV (+) group (p = 6e-10; *p* = 0.002; Figures 4G, H). We subsequently investigated the tumor mutation burden (TMB) in the groups C1 and C2 from TCGA database, but no statistically significant difference was found between them (*p* = 0.22, Supplementary Figures S2A, B).Tobacco use may contribute to the distribution of CafS, but we did not observe a significant difference between the smoking and non-smoking groups (TCGA, *p* = 0.76, GSE65858, *p* = 0.84, Supplementary Figures S3A, B). Moreover, we evaluated the CafS levels across all stages of head and neck squamous cell carcinoma (HNSCC), the results showed no statistical difference in CafS among different HNSCC stages (Figures 4I–L, *p* = 0.101, 0.194, 0.451, 0.483).

### High CafS changes the tumor immune microenvironment and is related to tumor associated macrophage (TAM)

**FIGURE 5:**
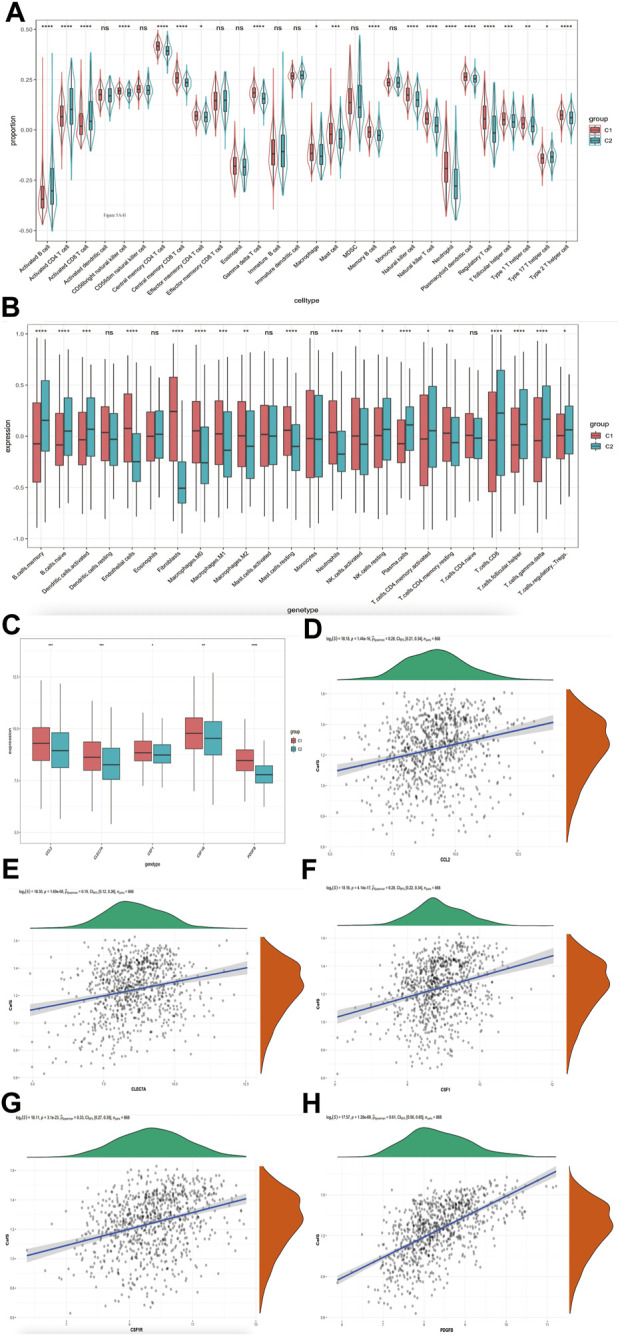
CafS changes the tumor immune microenvironment and is related to tumor associated macrophage (TAM) **(A)** Enrichment of 28 immune cell types infiltrating in the groups of CafS; combined database; the asterisk represents the different *p* values (* <0.05; ** <0.01; *** <0.001, **** <0.0001). **(B)** Boxplot of 24 immune cell types infiltrating by CIBERSORT algorithm in the groups of CafS; combined database; the asterisk represents the different *p* values (* <0.05; ** <0.01; *** <0.001, **** <0.0001). **(C)** Five genes’ expression of tumor associated macrophage (TAM) in the groups of CafS; the asterisk represents the different *p* values (* <0.05; ** <0.01; *** <0.001, **** <0.0001). **(D**–**H)** Correlation between CafS and CCL2, CLEC7A, CSF1, CSF1R and PDGFB (CafS and CCL2: r = 0.28, *p* = 1.44e-16; CafS and CLEC7A: r = 0.19, *p* = 1.69e-08; CafS and CSF1: r = 0.28, *p* = 4.14e-17; CafS and CSF1R: r = 0.33, *p* = 3.1e-23; CafS and PDGFB: r = 0.61, *p* = 1.28e-88).

We explored the relevence between the immune cell infiltration and CafS in the groups of C1 and C2. According to Bindea, G. et al. (Bindea et al., 2013) study, we calculated 28 immune cells value by the method of ssGSEA. Our results showed the proportion of activated CD4 T cell and activated CD8 T cell were significantly higher in the C2 cluster, on the contrary, the related macrophages infiltration was exceedingly higher in the C1 cluster (Figure 5A). We used CIBERSORT algorithm (Newman et al., 2015) to further detect the differential immune infiltration in the clusters C1 and C2. The results showed the expression of activated CD4 T cell and CD8 T cell were higher but macrophages (including M0, M1 and M2 status) were lower in C2 (Figure 5B). It reflected that high CafS may prevent immune cell cytotoxic effects but promote immune cell inflammatory effects in head and neck squamous cell carcinoma. M2 status macrophage often referred as tumor associated macrophage (TAM) which promote tumor growth, invasion, and metastasis (Ovais et al., 2019). So, we collected TAM markers from previous studies (Ngambenjawong et al., 2017; Jiang et al., 2022), they are CCL2, CLE7A, CSF1, CSF1R and PDGFB. We detected these TAM markers expression in the combined cohort, found all of them were significantly higher expression in high CafS group (Figure 4C). The correlation plots showed CafS was significantly positive correlated with these five TAM markers in combined database (CCL2: r = 0.28, *p* = 1.44e-16; CLEC7A: r = 0.19, *p* = 1.69e-08; CSF1: r = 0.28, *p* = 4.14e-17; CSF1R: r = 0.33, *p* = 3.1e-23; PDGFB: r = 0.61, *p* = 1.28e-88; respectively, Figures 5D–H).

### CafS changes hallmark signaling pathway and promotes the ability of tumor invasion

**FIGURE 6:**
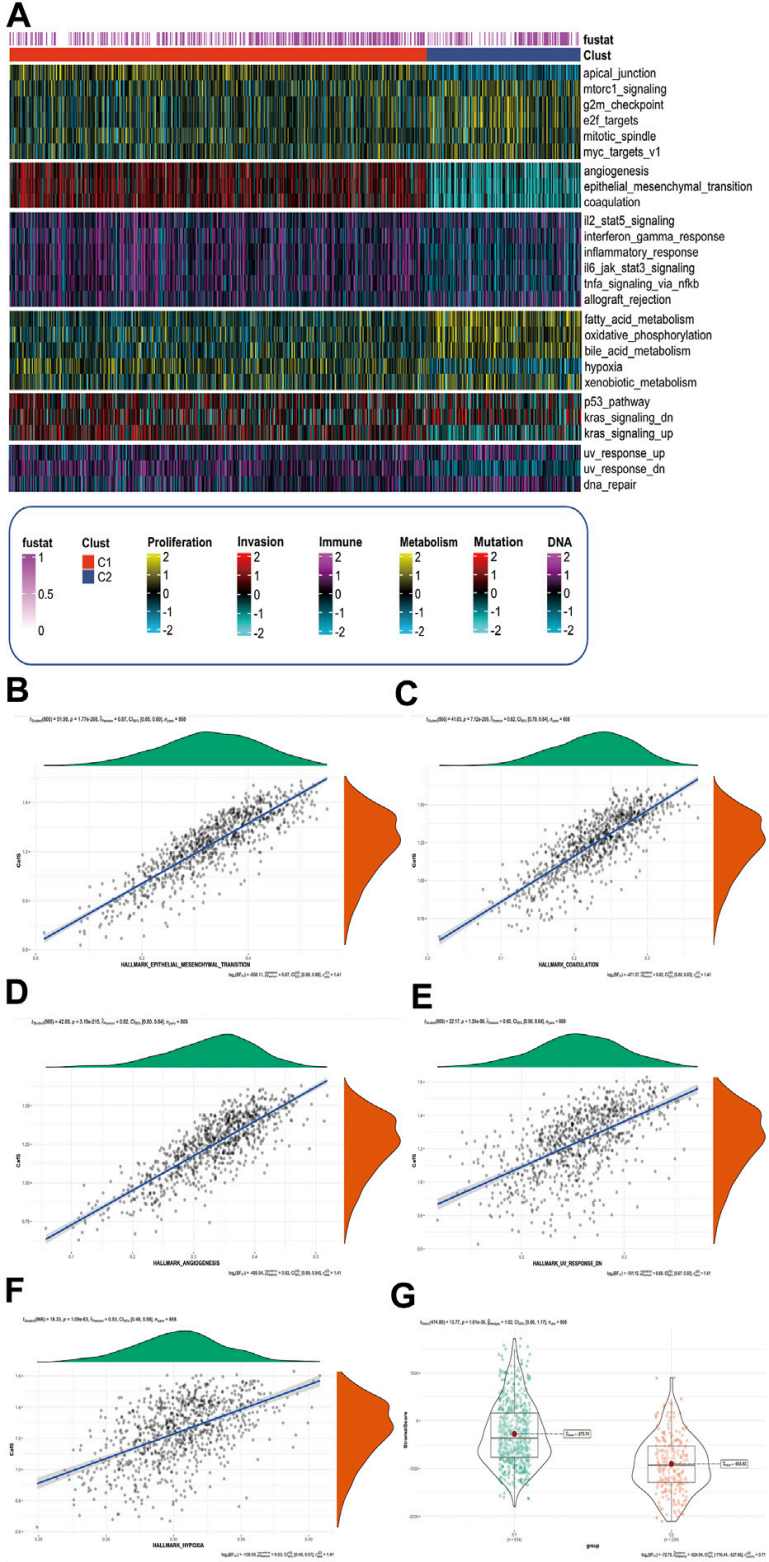
CafS changes hallmark signaling pathway and promotes the ability of tumor invasion **(A)** Complex-heatmap display the landscape in the combined cohort; the panel display the expression of hallmark signaling pathway involved in different CafS group; Proliferation, Invasion, Immune, Metabolism, Mutation and DNA represented six different pathway modules which arrange from the top down in complex-heatmap and they are labeled at the bottom of it; C1 represented high Cafs group and C2 represented low Cafs group; “fustat” means survival status, “1” means dead, “0” means alive. **(B**–**F)** Correlation between CafS and EMT, coagulation, angiogenesis, hypoxia, and uv-response-down pathway (CafS and EMT: r = 0.87, *p* = 1.77e-268; CafS and Coagulation: r = 0.82, *p* = 7.12e-209; CafS and Angiogenesis: r = 0.82, *p* = 3.19e-215; CafS and Hypoxia: r = 0.53, *p* = 1.09e-63; CafS and Uv-response-down: r = 0.60, *p* = 1.24e-86). **(G)** StromalScore in the groups of CafS; combined database; *p* = 1.61e-36.

We used GSVA method to analysis the characteristics of the associated signaling pathways in different CafS subtypes. The hallmark signaling pathway gene set was download from The Molecular Signatures Database (Liberzon et al., 2015) (MSigDB, https://www.gsea-msigdb.org/). We found high CafS in C1 had a remarkable enrichment in tumor invasion-related pathways such as angiogenesis, epithelial mesenchymal transition, and coagulation. In addition, we found there were different enrichments in pathways including tumor proliferation-related, tumor immune-related, tumor metabolism-related, tumor mutation-related, and tumor DNA damage-related (Figure 6A). We explored the relationship between CafS and tumor invasion-related pathways to further understand the mechanism of tumor process, we found CafS was significantly positive correlated with these tumor invasion-related, DNA damage-related and metabolism-related signaling pathways (EMT: r = 0.87, *p* = 1.77e-268; Coagulation: r = 0.82, *p* = 7.12e-209; Angiogenesis: r = 0.82, *p* = 3.19e-215; Hypoxia: r = 0.53, *p* = 1.09e-63; Uv-response-down: r = 0.60, *p* = 1.24e-86; respectively; Figures 6B–F). Fibroblasts contributed to a dominant component of the tumor stroma (Kalluri and Zeisberg, 2006), so, we used “ESTIMATE” R package to quantify the scores of stromal: the “StromalScore”. We found the StromalScore is profoundly higher in C1 than C2 group (Figure 6G).

### Verification of CAFs characteristics in single-cell RNA sequencing database

**FIGURE 7:**
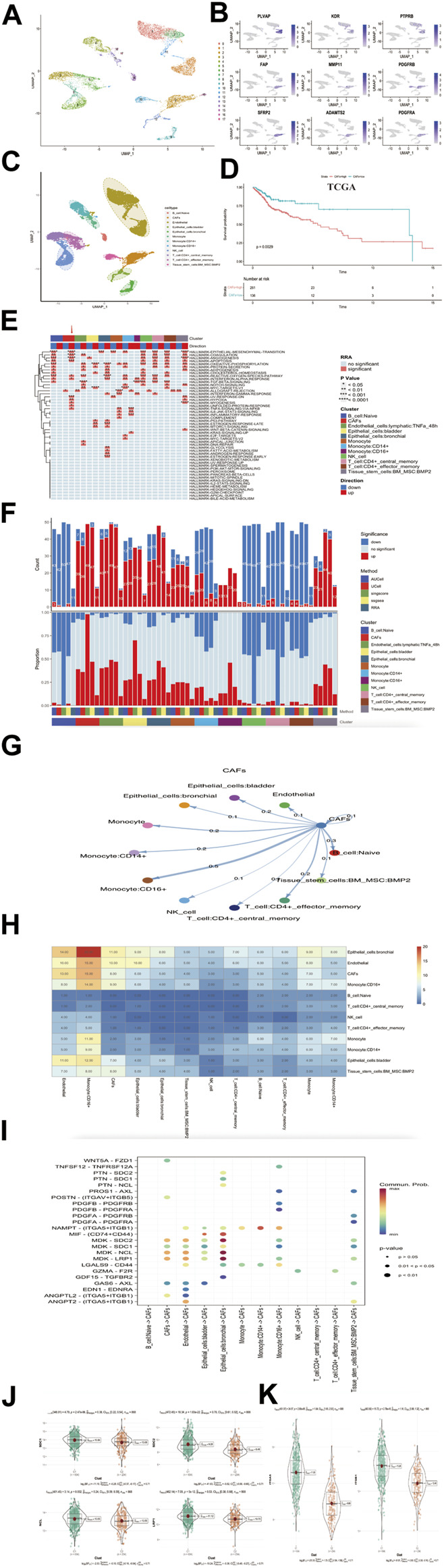
Verification of CafS clinical characteristic and biological function in single-cell RNA sequencing database **(A)** UMAP plot of selected 10244 single cells in tumor and non-immune stromal cells (CD45 negative). Different colors represent different cell types. **(B)** UMAP plot showed the expression of endothelial cell and cancer associated fibroblasts cell. **(C)** UAMP plot of selected 10244 single cells in tumor and non-immune stromal cells (CD45 negative). 18 cell clusters were divided into 12 cell types. **(D)** The Kaplan-Meier plot displays significant differences of survival time among the high-CAFs proportion and low-CAFs proportion in TCGA cohorts. Deconvolution method. High proportion group had worse overall time than low. **(E)** Display of the landscape of signaling pathways in different cell clusters; the panel display the hallmark signaling pathway involved in different cell clusters; Cluster (red module represent CAFs cell type), *p*-value and Direction of up or down are labeled at the right of plot; RRA represent significance. **(F)** Display of the landscape of gene set up-regulation or down-regulation in different cell clusters; Cluster (red module represent CAFs gene set), Method and Significance are labeled at the right of plot. **(G)** The differential cell–cell cellular communication shows CAFs weight coefficient between all cell types. **(H)** The heatmap of cell–cell cellular communication shows the counts of CAFs between all cell types. **(I)** Communication network of the significant ligand-receptor pairs between CAFs and other cell types, which contribute to the signaling from CAFs to Naive B cell, Endothelial Cell, Epithelial cell, Monocyte cell, NK cell, CD4^+^ T cell and Tissue stem cell subpopulations. Dot color reflects communication probabilities and dot size represents computed *p*-values. Empty space means the communication probability is zero. *p*-values are computed from one-sided permutation test. **(J)** SDC1, SDC2, NCL, LRP1 in the groups of Combined cohorts; *p* = 2.47e-06; *p* = 1.03e-22; *p* = 0.002; p = 5e-12. **(K)** ITGA5 and ITGB1 in the groups of combined cohorts; *p* = 2.88e-85; *p* = 2.78e-45.

We used R package “Seurat” (Butler et al., 2018) to analysis HNSCC single cell database. We selected CD45 negative as tumor and non-immune stromal cells to elucidate the heterogeneity of head and neck squamous. After quality control and filtering, we identified 10,244 cells from five head and neck squamous cell patients. We distinguished 18 distinct clusters based on a resolution value 0.5 (Figure 7A). We labeled cell types as endothelial cell (Endothelial, gene expression of PLVAP, KDR, PTPRB) and cancer associated fibroblasts cell (CAFs, gene expression of FAP, MMP11, PDGFRB, SFRP2, PDGFRA, ADAMTS2; Figure 7B). In addition to endothelial and cancer associated fibroblasts cell types classified above, we used “Single R” package to identify several other distinct clusters, they were b-cell Naïve, epithelial-cells bladder, epithelial-cells bronchial, monocyte, monocyte:CD14+, monocyte:CD16+, NK cell, CD4^+^ central memory T cell, CD4^+^ central effector T cell and tissue-stem-cells:BM_MSC:BMP2 (Figure 7C). To further understand the characteristics for the CAFs, we performed a deconvolution method (Wang et al., 2019) to calculate the bulk tissue proportion of CAFs in TCGA cohort with this single cell RNA sequencing database reference (Supplementary Table S1). Combining clinical data of TCGA-HNSC, we found higher CAFs proportion indicated significant poorer prognosis for head and neck squamous cell carcinoma (log rank *p* = 0.0029, Figure 7D). This result validated the high CAFs proportion may be identical to the high CafS as a prognostic indicator in HNSCC.

We next used irGSEA (https://github.com/chuiqin/irGSEA) package to analysis the associated signaling pathways in different cell clusters and focused more on CAFs type. The results exhibited CAFs cluster had a remarkable up-enrichment in tumor progression-related pathways such as angiogenesis, epithelial mesenchymal transition, coagulation, hypoxia and uv-response-down (Figure 7E), which were observed as the same to high CafS in group C1 (Figure 6A). We also detected CAFs cluster gene set expression, undoubtedly, CAFs cluster gene set was up-regulation (Figure 7F). These results illustrated up-regulation of CAFs genes could play a precondition role in activating specific signaling pathways such as angiogenesis, epithelial mesenchymal transition, coagulation, hypoxia, and uv-response-down, etc. This alternation influenced the tumor microenvironment and leaded to poorer prognosis in head and neck squamous cell carcinoma patients.

To further detect the enrichment of CAFs populations in HNSCC cells, we hypothesized that those CAFs populations might be functionally distinct across other different cell type. We hence performed the ligand–receptor-based cell-cell cellular cross-talk analysis (Jin et al., 2021). The plot showed the different weight coefficient distribution and counts frequency of CAFs to others cellular cross-talk (Figures 7G–H). These results suggested that HNSCC CAFs cells could preferentially reprogram and induce their specific functional status-likely explained by the specificity between genes’ differential expression, which could directly impact TME. We used the same method (Jin et al., 2021) to distinguish the signaling of ligand–receptor interactions network in HNSCC cells. We identified MDK and Nicotinamide phosphoribosyl transferase (NAMPT) ligand–receptor pairs contributing to the most communication from CAFs to each HNSCC cell type (Figure 7I). In combined bulk cohort, we further vitrificated high expression of these related ligand–receptor genes in high CafS group (Figures 7J–K). Therefore, these ligand–receptor pairs specifically enriched in HNSCC TME maybe provide a clue for targeted therapy.

### Construction and verification to the CAFs risk prediction model using machine learning methods

**FIGURE 8:**
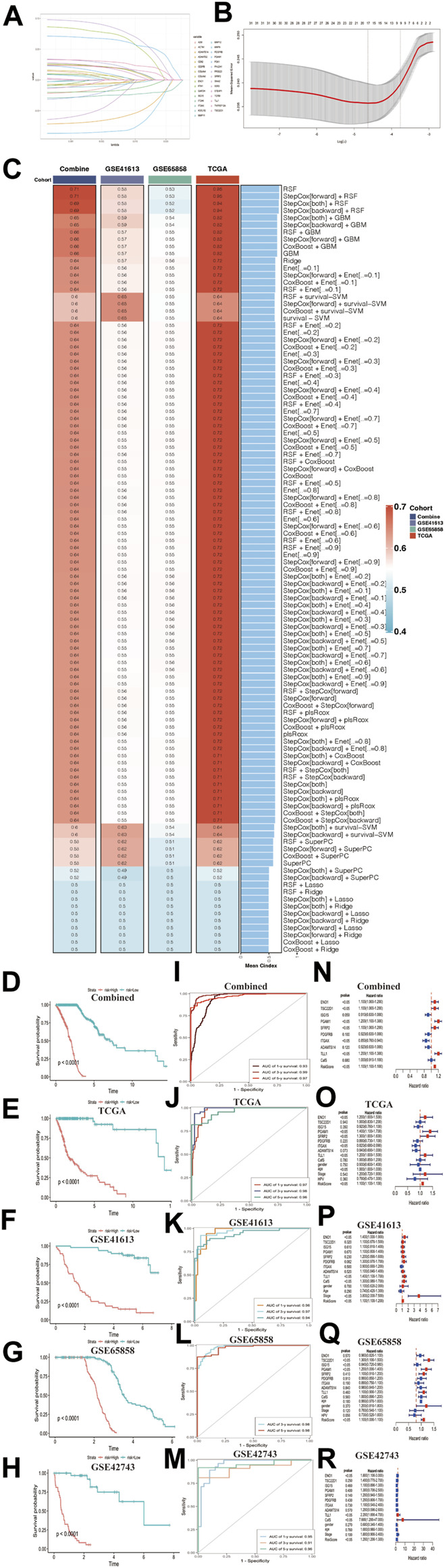
Construction and verification to the CAFs risk prediction model using machine learning methods **(A)** LASSO coefficient profiles of 31 cancer associated fibroblasts marker genes in Combined cohort. **(B)** 1000‐time cross‐validation for tuning parameter selection in the LASSO model; Combined cohort. **(C)** A total of 107 kinds of prediction models *via* machine learning and further calculated the C-index of each model across training and all validation cohorts. **(D**–**H)** Kaplan–Meier curves of overall survival according to the median risk score in Combined, TCGA-HNSC, GSE41613, GSE65858, and GSE42743 external validation cohorts. All log-rank *p* < 0.0001. **(I**–**M)** Time-ROC value in Combined, TCGA-HNSC, GSE41613, GSE65858, and GSE42743 external validation cohorts. **(I)** Combined, AUC one-three-five years = 0.93, 0.99, 0.97. **(J)** TCGA-HNSC, AUC one-three-five years = 0.97, 0.98, 0.96. **(K)** GSE41613, AUC one-three-five years = 0.96, 0.97, 0.94. **(L)** GSE65858, AUC one-three years = 0.98. **(M)** GSE42743, AUC one-three-five years = 0.95, 0.91, 0.96. **(N**–**R)** Multivariate Cox regression of risk score regarding to OS in the Combined, TCGA-HNSC, GSE41613, GSE65858 and GSE42743. Statistic tests: two-sided Wald test. Data are presented as hazard ratio (HR) ± 95% confidence interval [CI].

Several studies have proved that CAFs genes were biomarker for prognostic in many types of cancer (Wen et al., 2019; Li et al., 2021a; Shelton et al., 2021). So, we used the Lasso algorithm to filter the most candidate prognostic CAFs genes from the classification model (Figures 8A, B). Considering the convenience for the future clinical testing, we selected 9 CAFs genes (including ENO1, TSC22D1, ISG15, PGAM1, SFEP2, PDGFRB, ITGAX, ADAMTS14 and TLL1) and CafS to construct risk model. We set TCGA-HNSC database as training cohort; the combined cohort, GSE65858, and GSE41613 cohorts as validation datasets. GSE42743 cohort was used to external verification. In TCGA-HNSC cohort, we first fitted 107 kinds of prediction models *via* the 10 machine learning algorithms and further calculated the C-index value of each model across all validation datasets (Figure 8C). Interestingly, the optimal training model with the highest C-index value (0.95) was designed by random survival forest (RSF) algorithm, and this model also present highest average C-index value (0.61) in all validation cohorts (Figure 8C). Next, a risk score for each patient was calculated using the “predict” function in this RSF model, according to their median risk score, all patients were divided into high- and low-risk groups. The Kaplan–Meier curve showed patients in the high-risk group had significantly dismal overall survival (OS) compared to the low-risk group in the TCGA-HNSC training dataset and four validation datasets (Figures 8D–G, all log rank *p* < 0.0001). The trend of this finding was validated in the external cohort GSE42743 using the same method (Figure 8H, log rank *p* < 0.0001). Time receiver operating characteristic (ROC) method was applied to verify the sensitivity and specificity to the risk model. As we had expected, the results demonstrated that all datasets had remarkably delight Time-ROC values (combined, AUC one-three-five years = 0.93, 0.99, 0.97; TCGA-HNSC, AUC one-three-five years = 0.97, 0.98, 0.96; GSE41613, AUC one-three-five years = 0.96, 0.97, 0.94; GSE65858, AUC one-three years = 0.98; GSE42743, AUC one-three-five years = 0.95, 0.91, 0.96; Figures 8I–M). Those results indicated this CAFs related risk model had considerably predictive significance. Multivariate Cox regression demonstrated that risk score remained statistically significant (all *p*-value < 0.05) in all cohorts after adjusting for available clinical traits, such as age (less than 60 vs. be equal or greater than 60); gender (Female vs. Male); stage and HPV status, the results suggested that risk score is an independent predict factor for overall survival (Figures 8N–R).

### Relationship between CafS classification pattern and CAFs risk score model

**FIGURE 9:**
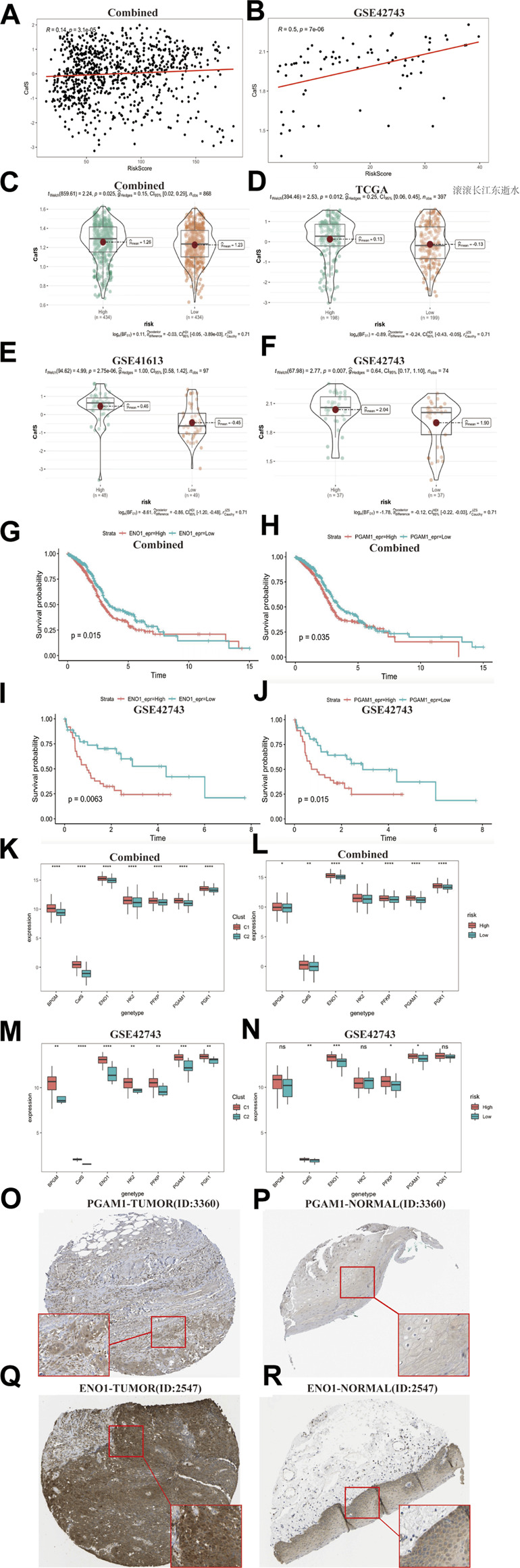
Relationship between CafS classification pattern and CAFs risk score model **(A**, **B)** The correlation between CafS and risk score in the combined and external cohort GSE42743; Combined, r = 0.14, *p* = 3.1e-05; GSE42743, r = 0.5, p = 7e-06. **(C**–**F)** CafS in the high and low risk groups in the combined, TCGA-HNSC, GSE41613 and GSE42743 cohort *p* = 0.025, 0.012, 2.57e-06, 0.007, respectively. **(G**–**J)** Kaplan–Meier curves of overall survival according to the median of ENO1 and PGAM1 expression in the Combined and GSE42743 external validation cohorts. Combined, ENO1 and PGAM1, log-rank *p* = 0.015, 0.035, respectively. GSE42743, ENO1 and PGAM1, log-rank *p* = 0.0063, 0.015, respectively. **(K**–**N)** ENO1, PGAM1, HK2, PFKP, BPGM, PGK1 and CafS expression in the CafS classification model and risk score model; Combined and external GSE42743 cohorts; the asterisk represents the different *p* values (* <0.05; ** <0.01; *** <0.001, **** <0.0001). **(O**–**R)** Immunohistochemical of PGMA1 and ENO1 in the matched tumor and normal side.

As the results showed above: the high CafS and CAFs related high-risk score both indicate worse survival, we assumed that those HNSCC populations with high-risk score seem to combine with high CafS. Hence, we generated a correlation map which showed CafS was significantly positive correlated with the risk score in the combined and external validation cohort (r = 0.19; *p* = 3.1e-05; r = 0.5, p = 7e-06; Figures 9A, B). We further calculated the CafS in the groups of risk model and found that the model risk score might fit CafS distribution in the combined and validation cohorts (combined, *p* = 0.025; TCGA-HNSC, *p* = 0.012; GSE41613, *p* = 2.75e-06; GSE42743, *p* = 0.007; Figures 9C–F). These results confirmed our hypothesis that there is a certain degree of interaction between CafS and risk score thereby bringing dark survival to HNSCC patients. In the Multivariate Cox regression model, we found ENO1 and PGAM1were glycolysis enzyme markers (Huang et al., 2022a; Yang et al., 2022) may contribute to bad outcomes. According to the gene median expression, the ENO1 and PGAM1 were significant predictors in both combined and validation cohorts (combined, ENO1, log-rank *p* = 0.015, PGAM1, log-rank *p* = 0.035; GSE42743, ENO1, log-rank *p* = 0.0063, PGAM1, log-rank *p* = 0.015; Figures 9G–J). As the results described above, we infer that high CafS and risk score could induce glycolysis reprograming, we hence collected other four crucial glycolysis enzyme biomarkers from previous study (Warmoes and Locasale, 2014), they are BPGM, HK2, PFKP and PGK1. In the CafS classification model, the results showed these genes’ expression presented higher in the group of C1 (Figures 9K, L). In the risk model, glycolysis enzyme marker genes expression also was observed increased trend in the combined cohort, but not all were observed high expression in the GSE42743 external validation cohort, possibly due to the sample size (Figures 9M, N). Based on matched tumor and normal tissues from the patients in Human Protein Atlas, we found both PGAM1 and ENO1 were up-regulation in tumor side (Figures 9O–R). The qPCR assay also validated that PGAM1 and ENO1 were over expression in HNSCC cell lines compare to nasopharyngeal epithelial cell line (Supplementary Figures S4A–D). Hence, the above results provide us a clue for targeted glycolysis reprograming therapy might make breakthroughs in CAFs treatment.

## Discussion

At present, a lot of studies only use TCGA or single GSE cohort as data sources to analysis malignant tumor, which are short of sample size and beyond to the accuracy and effectiveness for medical practice. In our study, we collected 868 cases to explore the molecular actions of CAFs in head and neck squamous cell carcinoma. We constructed a robust CAFs related classification and score system using 31 CAFs marker genes, which could effectively predict the prognosis of the HNSCC patients. We found there were remarkable discrepancy in clinical and biological characteristics such as HPV status and TAMs among different CafS clusters. HPV-negative head and neck tumors patients was confirmed with terrible prognoses (Johnson et al., 2020). In our study, high CafS patient was more likely to be HPV-negative, indicated that CafS could exert adverse impact on clinical outcomes. Tumor associated macrophages marker genes played a crucial role in tumor process, for example, high expression CCL2 in macrophages could promote HNSCC invasion and metastasis (Ling et al., 2022). CLEC7A also called Dectin1, Daley, D. et al. (Daley et al., 2017) found it activated macrophages and promotes pancreatic ductal adenocarcinoma progression. CSF1/CSF1R signaling axis had been proved induced macrophages to M2 polarization and promoted tumor growth and lung metastasis (Fujiwara et al., 2021). PDGFB as a platelet activation factor for promoting tumor metastasis by recruitment of TAMs (Yang et al., 2016). In our study, we showed these five TAMs markers not only were exceedingly high expression in C1 group but closely associated with CafS, these results illustrated high CafS was associated with abundant M2 macrophages enrichment and provided us an expanded knowledge for the CAFs genes’ role in the tumor microenvironment.

Analysis for the associated signaling pathways in different CafS groups revealed interesting findings. First, high CafS represented extensively activation in pathways such as angiogenesis, epithelial mesenchymal transition, and coagulation, all these pathways enhanced tumor cell malignancy (Nash et al., 2001; Zhang et al., 2021a; Huinen et al., 2021). UV-response-down pathway was a process that organism undergo UV-B or UV-A radiation may generate genomic mutations and instability leading to tumorigenesis (Tan et al., 2019). In this study, we found uv-response-down was up-regulation in C1 group, it broadened our horizons of CAFs carcinogenesis. Hypoxia pathway could activate multiple genes’ expression which participate in iron metabolism, glucose transport, cell proliferation thereby resulting in poor prognosis of treatment (Nordgren and Tavassoli, 2011). Our result showed high CafS aggravate head and neck squamous cell carcinoma hypoxic condition and displayed a remarkable correlation between these five tumor-related signaling pathways and CafS, which enhanced our comprehension for CAFs promoting HNSCC proliferation and metastasis. In addition, high CafS was characterized by leading stromal score, hence are more likely to lead to tumor capped by extracellular matrix and induce immunosuppression (Buchheit et al., 2014).

Single cell RNA sequencing revealed head and neck squamous cell complexity and heterogeneity. We identified CAFs clusters and other 11 distinct cell types. Similar to the bulk tissue sequencing results, our results validated CAFs population possess the characteristics of strong cancer-promoting signatures, it indicated angiogenesis, epithelial mesenchymal transition, coagulation, uv-response-down and hypoxia pathways were up-regulated in this cell cluster. The up regulation of CAFs gene set profile contribute to the activation of these related signaling pathways. Moreover, the deconvolution result showed high CAFs proportion, just like high Cafs, robustly correlated with poor survival in TCGA cohort, suggesting a prospective adoption to CAFs biomarkers for HNSCC treatment.

Comprehensive investigations of intercellular communications are essential for understanding interactions and spatial proximity between CAFs and other cell types. Midkine (MDK) belong to a group of heparin-binding growth factors that has been shown to have pleiotropic functions in various biological processes during development and disease (Cui and Lwigale, 2019). It has been reported to overlap with the expression of SCD1 and LRP1 and promote epidermal growth factor receptor (EGFR) signaling by interacting with surface nucleolin (NCL) in hypoxic condition (Cui and Lwigale, 2019; Kinoshita et al., 2020). In addition, overexpression of SDC1 and SDC2 were associated with more aggressive in prostate cancer and MDK-LRP1 will induce the differentiation of immunosuppressive macrophages (Zhang et al., 2021b; Santos et al., 2021). In our study, we first identified those MDK related ligand-receptor pairs as the dominant signaling facilitate to the cellular cross-talk between CAFs and other cell types. We further contextualize this finding in our combined bulk cohort, thus those ligand-receptor analysis of the putative interactions displayed here can be pursued further to better understand the ecosystem cultivated by intercellular communication in the HNSCC tumor microenvironment. Nicotinamide phosphoribosyl transferase (NAMPT) played a crucial role in cancer cell metabolism, often overexpressed in tumor tissues and was an effective target for antitumor treatments (Garten et al., 2015). NAMPT inhibitor was proved to effectively repress cell growth in head and neck squamous cell carcinoma (Cai et al., 2022). In our study, we revealed the extensive enrichment of NAMPT ligand-receptor pair (ITGA5, ITGB1) communication, we also found ITGA5 and ITGB1 were overexpressed in high CafS group, thus providing an explanation for the complex pro tumorigenic mechanism of CAFs.

With the expression profiles of these CAFs genes, we developed an integrative pipeline to construct a predictive model according to the CafS classifier. We first used Lasso algorithm to screen the contents of model container. In total, 9 CAFs related genes and 107 kinds of models were fitted to the training datasets *via* machine learning. Further validation in independent cohorts revealed that the optimal model was random survival forest (RSF). In contrast to the former studies, the advantage of this model with consensus performance on the prognosis of HNSCC is based on a variety of machine learning algorithms and their combinations, which further make this model more convincing to accurate prognosis. TIME-ROC curve suggested that risk score calculated by this model maintained the high precision and high stable performance in all datasets, which indicated great potential for the future clinical application using this risk score. In addition, compared to the conventional tools such as age, gender, stage and HPV status for evaluating clinical outcomes, the risk score signature worked independently of these factors and had significantly superior efficiency in predicting prognosis in training and validation cohorts. We also reviewed previous published HNSCC-related risk models which including different genes’ combination (Liu et al., 2020; Li et al., 2021b; He et al., 2021; Wang et al., 2022a; Huang et al., 2022b; Wang et al., 2022b; Chen et al., 2022; Chi et al., 2022; Du et al., 2022; Han et al., 2022; Peng et al., 2022), among these, none of them presented better AUC value performance than our model. Therefore, our risk score signature could be a promising surrogate for evaluating the prognosis of HNSCC in clinical practice.

Combining the Multivariate Cox regression model and Kaplan–Meier curve, the result revealed glycolysis enzyme biomarkers ENO1 and PGAM1 might be important predictors of overall survival in HNSCC. They have been verified to promote cancer cell proliferation and progression (Ishikawa et al., 2014; Qiao et al., 2021). Another study proved CAFs could secrete cytokines and chemokines thus triggering mobilization of glycogen in cancer cells and induce glycolysis reprograming, this CAFs-mediated glycolysis reprograming then results in the invasion and metastasis enhanced in ovarian cancer (Curtis et al., 2019). In our study, we found ENO1 and PGAM1 both were up-regulation in C1 and high-risk score group. In addition, the same trend was observed in the other four glycolysis enzyme markers, and we validate ENO1 and PGAM1 were overexpression in matched tumor part compared to normal side. Hence, we suggested CAFs could dominate the tumor metabolism microenvironment by inducing glycolysis reprograming in head and neck squamous cell carcinoma. To this end, glycolysis inhibitors present a hopeful method to improve CAFs targeted therapeutic strategy.

Although these promising findings were detected in this study, we acknowledge some limitations. For example, we should verify our results using fresh tumor samples, further biological experiment, including cell and molecular assays need to validate the findings of this study. In addition, we conducted a retrospective study, and future validation should be performed in a prospective multicenter cohort.

In conclusion, we constructed a classification system to distinguish the CAFs-related subtype in head and neck squamous cell carcinoma. We observed the potential mechanism of carcinogenesis to CAFs genes and revealed unique possibilities to target glycolysis pathways to enhance CAFs targeted therapy. We developed an unprecedentedly stable and powerful risk score for assessing the prognosis. Our study contributes to the understanding of the CAFs microenvironment complexity in patients with head and neck squamous cell carcinoma and serves as a basis for future in-depth CAFs gene clinical exploration.

## Data Availability

Publicly available datasets were analyzed in this study. This data can be found here: https://www.jianguoyun.com/p/DVCMPk0QuK-dChj-4ugEIAA.

## Supplementary material

Supplementary Figure S1
**(A–C)** The tabular formats of clinical sample information of TCGA, GSE65858 and GSE41613 cohorts.

Supplementary Figure S2
**(A)** The tumor mutation waterfall plot. **(B)** Tumor mutation burden (TMB) in the groups C1 and C2 from TCGA database, *p* = 0.22.

Supplementary Figure S3
**(A–B)** CafS distribution in the smoking and no smoking groups; TCGA (*p* = 0.76) and GSE65858 (*p* = 0.84).

Supplementary Figure S4
**(A**–**D)** PGAM1 and ENO1 expression in normal nasopharyngeal epithelial cell line (NP69) and HNSCC cell lines (CAL-27 and FaDu), quantitative real-time PCR assay, **p* < 0.05; ***p* < 0.01; ****p* < 0.001; *****p* < 0.0001.
