# Supplementary figures and images for: Clustering and machine learning-based integration identify cancer associated fibroblasts genes’ signature in head and neck squamous cell carcinoma
Source: Front Genet. 2023 Mar 30;14:1111816. doi: 10.3389/fgene.2023.1111816 (PMC10098459; doi:10.3389/fgene.2023.1111816)

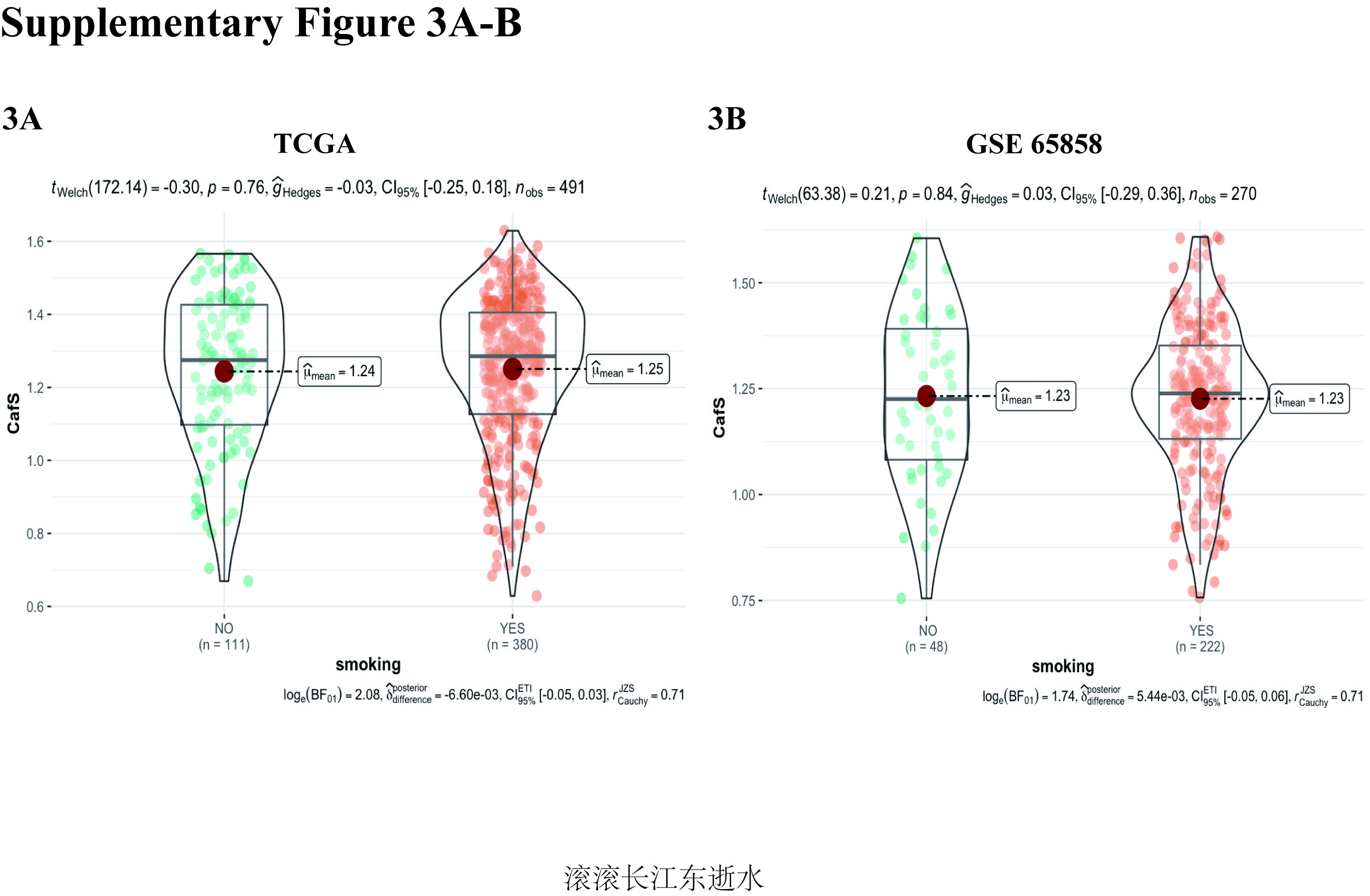

Supplement: Supplementary file 1 [file Image3.jpeg]

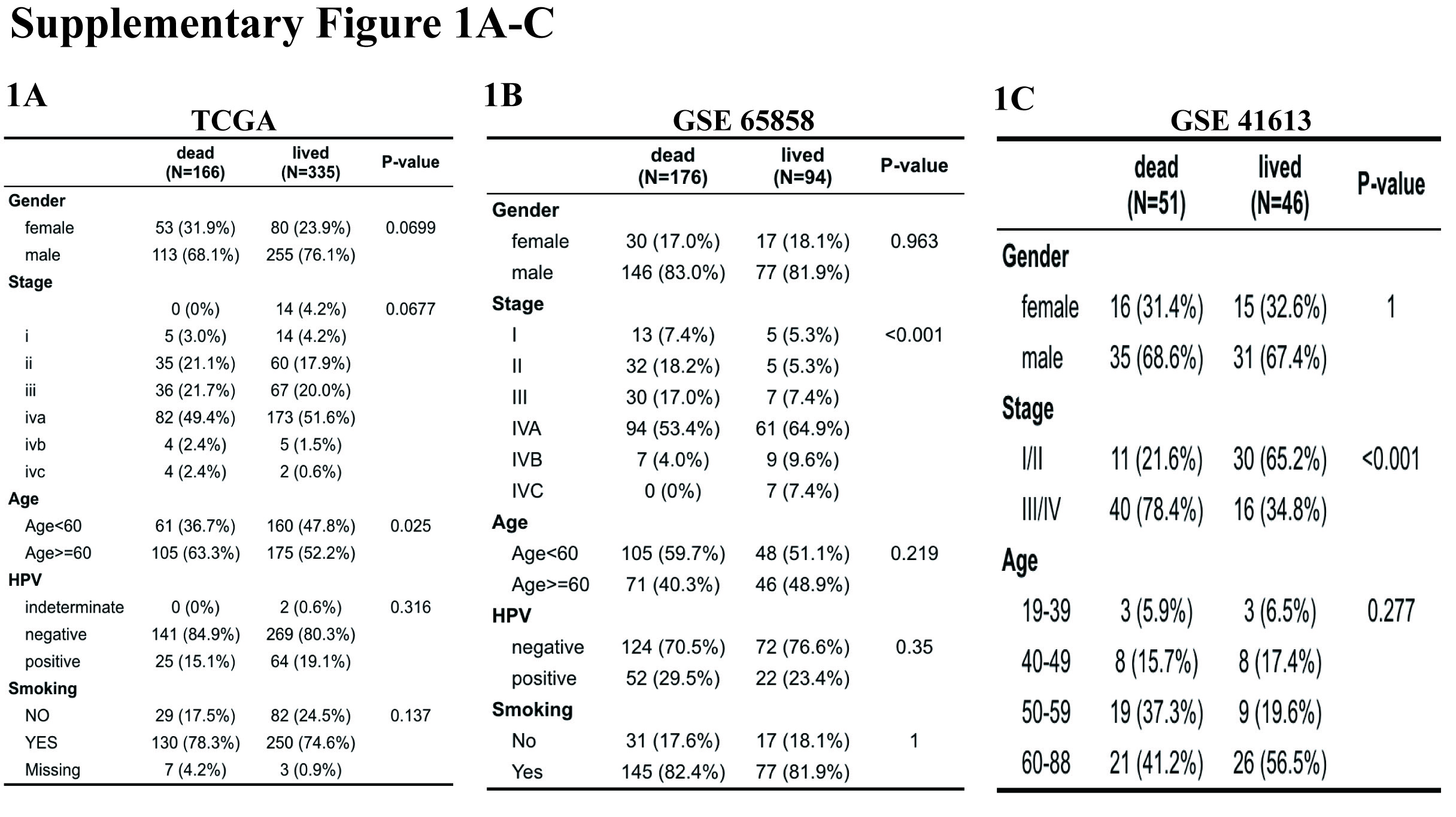

Supplement: Supplementary file 2 [file Image1.jpeg]

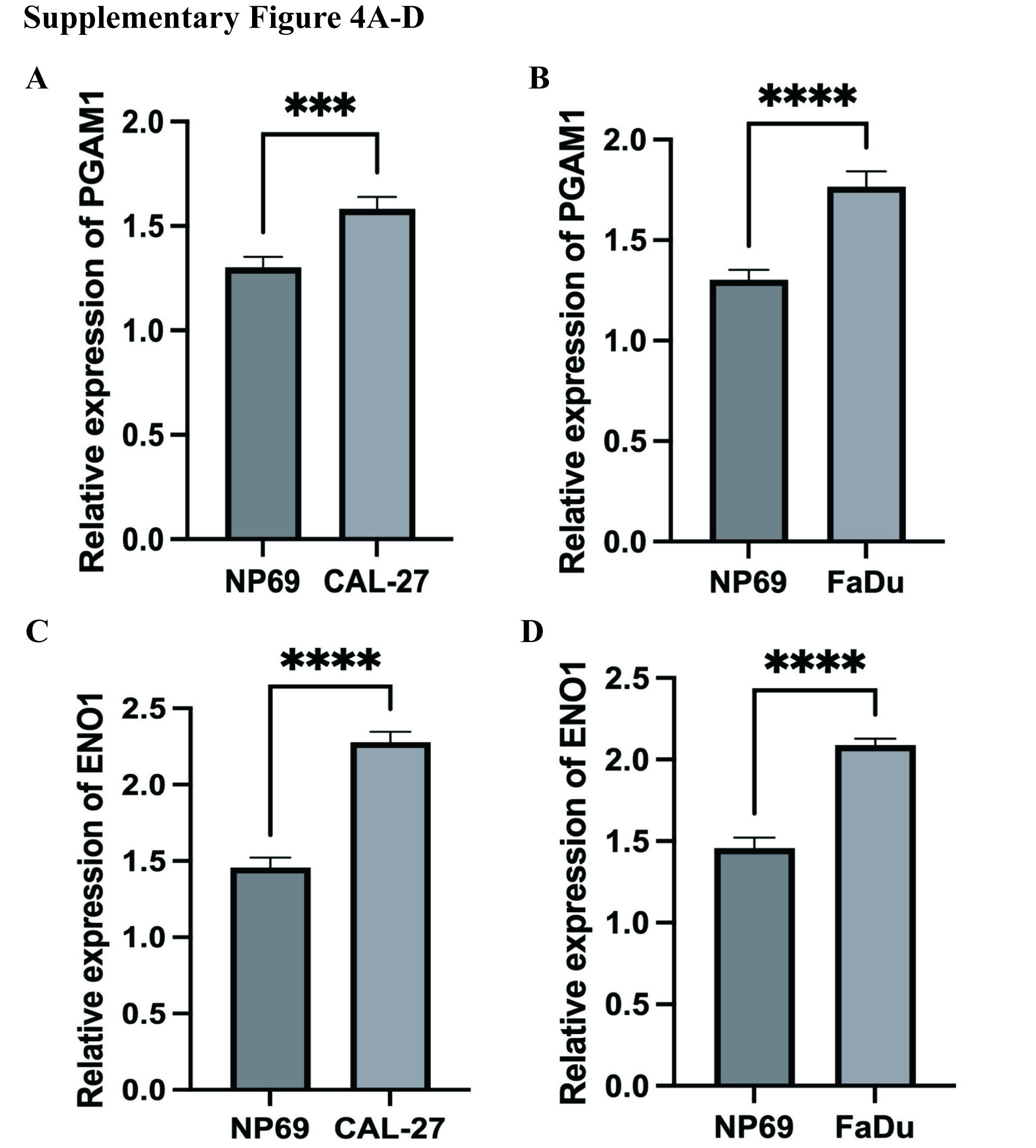

Supplement: Supplementary file 3 [file Image4.jpeg]

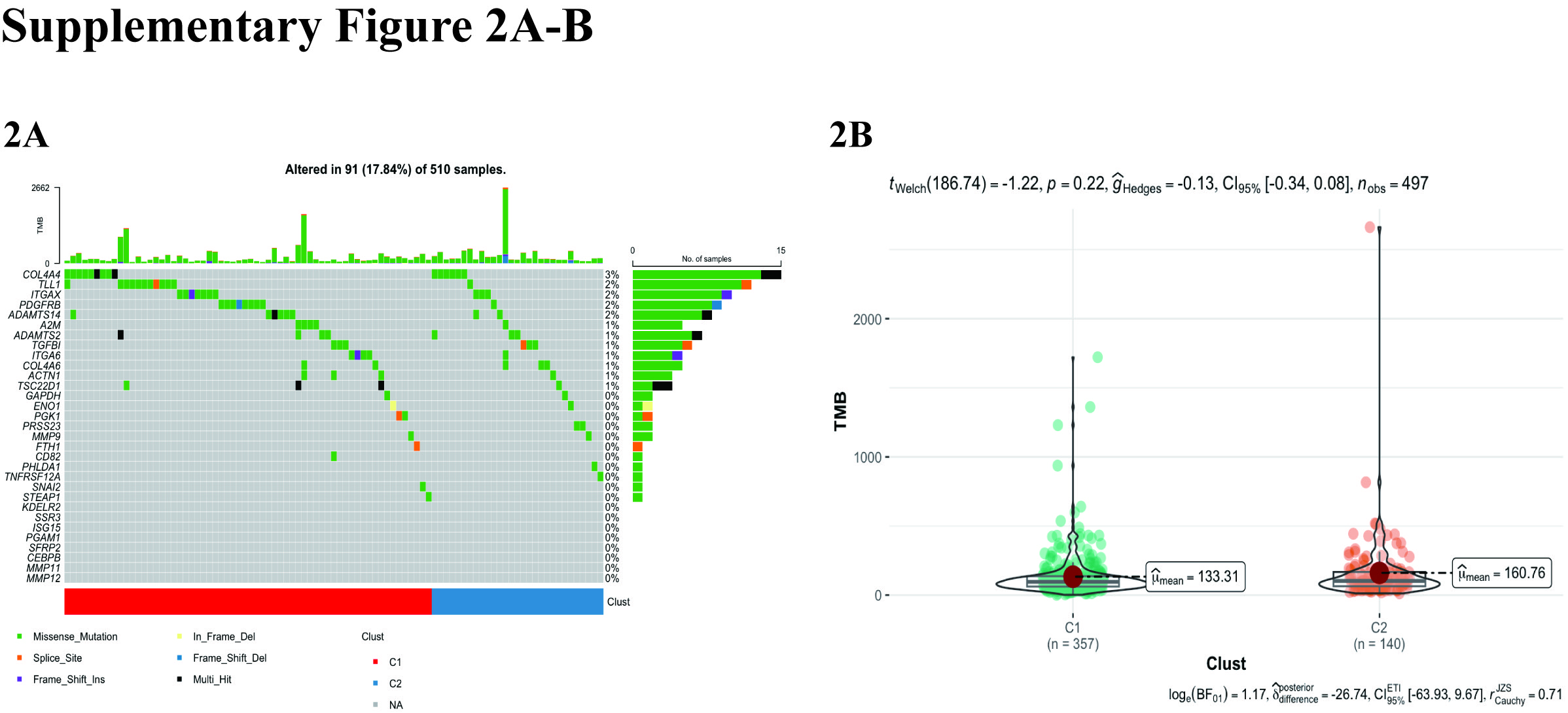

Supplement: Supplementary file 4 [file Image2.jpeg]
