# Supplementary material for: Clustering and machine learning-based integration identify cancer associated fibroblasts genes’ signature in head and neck squamous cell carcinoma
Source: Front Genet. 2023 Mar 30;14:1111816. doi: 10.3389/fgene.2023.1111816 (PMC10098459; doi:10.3389/fgene.2023.1111816)
Supplement: Supplementary file 5 [file Table1.DOC]

**Supplementary Table1: MuSic deconvolution result**

|  | Enthodelial | CAFs |
| --- | --- | --- |
| TCGA-D6-6515 | 0.00038439 | 0.0005748 |
| TCGA-CV-7180 | 0.00036747 | 0.00044619 |
| TCGA-CV-7183 | 0.00043043 | 0.00054178 |
| TCGA-CV-A6JN | 0.00012021 | 9.87E-05 |
| TCGA-CQ-7069 | 0.0006608 | 0.00090264 |
| TCGA-CQ-6219 | 0.00032124 | 0.00044064 |
| TCGA-IQ-A6SH | 0.00020011 | 0.00024408 |
| TCGA-F7-A61V | 7.58E-05 | 8.19E-05 |
| TCGA-HD-A6I0 | 0.00049251 | 0.00053318 |
| TCGA-CN-6992 | 3.89E-05 | 6.95E-05 |
| TCGA-BA-5556 | 0.00011888 | 0.00013033 |
| TCGA-CV-7245 | 0.00018836 | 0.0002818 |
| TCGA-UF-A7JV | 7.65E-05 | 6.89E-05 |
| TCGA-CV-5440 | 0.00027633 | 0.00030602 |
| TCGA-UF-A7JO | 6.45E-05 | 8.62E-05 |
| TCGA-CV-5443 | 0.00035973 | 0.00050107 |
| TCGA-P3-A5Q5 | 2.04E-05 | 1.04E-05 |
| TCGA-CN-A498 | 0.00022361 | 0.00026147 |
| TCGA-CX-7082 | 0.00039591 | 0.00054802 |
| TCGA-CV-7432 | 0.00014141 | 9.94E-05 |
| TCGA-CV-7248 | 4.31E-05 | 5.75E-05 |
| TCGA-KU-A66T | 0.00030707 | 0.00034191 |
| TCGA-P3-A5Q6 | 7.09E-05 | 0.00011391 |
| TCGA-CN-5374 | 2.75E-05 | 2.68E-05 |
| TCGA-IQ-A61J | 0.00037941 | 0.00054431 |
| TCGA-CV-A45Y | 0.00028609 | 0.0004083 |
| TCGA-P3-A6T0 | 9.35E-05 | 0.00014224 |
| TCGA-CN-4740 | 3.22E-05 | 5.32E-05 |
| TCGA-CN-5370 | 3.26E-05 | 5.34E-05 |
| TCGA-CN-6024 | 0.00042877 | 0.00061526 |
| TCGA-CR-7397 | 7.55E-05 | 0.00012705 |
| TCGA-QK-AA3J | 4.71E-05 | 4.59E-05 |
| TCGA-BA-A6DI | 0.00019026 | 0.00023742 |
| TCGA-CV-7413 | 0.00025573 | 0.00040138 |
| TCGA-CR-7377 | 0.00032188 | 0.00049746 |
| TCGA-D6-6823 | 6.83E-05 | 8.09E-05 |
| TCGA-CV-7253 | 0.00013085 | 0.00013591 |
| TCGA-CV-7238 | 0.00066833 | 0.00078965 |
| TCGA-CV-6942 | 0.0001125 | 0.00018189 |
| TCGA-BA-A6DA | 0.00049283 | 0.00054562 |
| TCGA-MT-A67D | 0.00028872 | 0.00040039 |
| TCGA-CQ-5323 | 0.0001658 | 0.00027564 |
| TCGA-CR-7390 | 0.00011583 | 0.00016194 |
| TCGA-UF-A7JK | 0.00010021 | 0.00011779 |
| TCGA-DQ-7591 | 0.0001085 | 0.00010576 |
| TCGA-CN-5358 | 7.00E-05 | 0.00012405 |
| TCGA-CV-6945 | 0.00018318 | 0.0002254 |
| TCGA-CQ-6218 | 0.00033689 | 0.00044468 |
| TCGA-CV-6943 | 0.00066113 | 0.00041651 |
| TCGA-CN-4737 | 0.00049275 | 0.00070135 |
| TCGA-QK-A6V9 | 4.77E-05 | 3.48E-05 |
| TCGA-CR-6472 | 0.0003336 | 0.00041717 |
| TCGA-UF-A7JH | 7.68E-05 | 0.00012373 |
| TCGA-BA-4077 | 0.00035534 | 0.00043556 |
| TCGA-CV-7411 | 0.00073553 | 0.00114143 |
| TCGA-MT-A67A | 0.00026342 | 0.00026117 |
| TCGA-F7-8489 | 0.0003733 | 0.00051417 |
| TCGA-DQ-5625 | 0.00030229 | 0.00039192 |
| TCGA-CV-6950 | 0.00011926 | 0.00019063 |
| TCGA-P3-A6T7 | 0.00012759 | 0.00014697 |
| TCGA-UF-A718 | 5.41E-05 | 0.00010403 |
| TCGA-QK-A6VB | 4.35E-05 | 7.75E-05 |
| TCGA-CV-6938 | 1.48E-05 | 2.87E-05 |
| TCGA-CV-7250 | 0.00014679 | 0.00017559 |
| TCGA-CV-6959 | 0.0001236 | 0.00019892 |
| TCGA-HD-7832 | 0.00012564 | 0.00021849 |
| TCGA-CV-A45X | 0.00034317 | 0.00038058 |
| TCGA-CN-A6UY | 4.14E-05 | 3.99E-05 |
| TCGA-CQ-6222 | 0.00015299 | 0.00012776 |
| TCGA-MZ-A7D7 | 3.25E-05 | 4.57E-05 |
| TCGA-CQ-5329 | 2.92E-05 | 8.83E-05 |
| TCGA-CV-5973 | 0.00021503 | 0.00029388 |
| TCGA-BA-5555 | 0.00014167 | 0.0001905 |
| TCGA-CV-6935 | 0.00046567 | 0.00049092 |
| TCGA-HD-8635 | 0.00043086 | 0.00060066 |
| TCGA-CR-6487 | 0.00026639 | 0.00030721 |
| TCGA-CN-6018 | 0.00035706 | 0.00051165 |
| TCGA-CR-6484 | 0.0003771 | 0.0005674 |
| TCGA-CV-7425 | 0.00016135 | 0.00018473 |
| TCGA-BB-A5HU | 5.04E-05 | 6.33E-05 |
| TCGA-HD-7831 | 7.05E-05 | 0.00015367 |
| TCGA-CR-7364 | 9.39E-05 | 0.00015865 |
| TCGA-CN-A63W | 6.51E-05 | 9.50E-05 |
| TCGA-D6-A6EK | 0.00010533 | 6.94E-05 |
| TCGA-BA-5557 | 8.83E-05 | 0.00016674 |
| TCGA-BA-6869 | 6.17E-05 | 9.05E-05 |
| TCGA-CR-6477 | 8.56E-05 | 0.00013679 |
| TCGA-UF-A71B | 0.00033556 | 0.00044607 |
| TCGA-H7-A6C5 | 0.00014896 | 0.00016278 |
| TCGA-CV-6933 | 0.00157684 | 0.00140989 |
| TCGA-QK-A6IF | 1.87E-05 | 1.42E-05 |
| TCGA-DQ-7588 | 9.54E-05 | 7.94E-05 |
| TCGA-BA-6873 | 0.00032035 | 0.00043919 |
| TCGA-CV-5442 | 0.00016859 | 8.43E-05 |
| TCGA-CR-7392 | 0.00044366 | 0.00067163 |
| TCGA-UF-A71A | 4.94E-05 | 6.30E-05 |
| TCGA-CN-A642 | 2.99E-05 | 5.37E-05 |
| TCGA-CV-A45U | 0.0003714 | 0.00046722 |
| TCGA-CN-6012 | 7.29E-05 | 0.00011532 |
| TCGA-CV-A45W | 0.00026219 | 0.0003275 |
| TCGA-CV-A6K0 | 0.00027997 | 0.00041619 |
| TCGA-CN-6022 | 1.73E-05 | 2.86E-05 |
| TCGA-CN-A6V3 | 9.81E-05 | 0.00013031 |
| TCGA-QK-A8Z7 | 2.55E-05 | 2.83E-05 |
| TCGA-CN-A497 | 0.00040438 | 0.00053663 |
| TCGA-F7-A50G | 0.00031692 | 0.0004078 |
| TCGA-IQ-A61I | 4.37E-05 | 4.46E-05 |
| TCGA-CR-7373 | 0.00010148 | 0.00014092 |
| TCGA-CN-A63T | 3.62E-05 | 4.71E-05 |
| TCGA-CN-6019 | 2.65E-05 | 4.27E-05 |
| TCGA-CR-7386 | 8.74E-05 | 0.00019016 |
| TCGA-HD-A6HZ | 0.00033527 | 0.0004782 |
| TCGA-T3-A92M | 1.18E-05 | 3.02E-05 |
| TCGA-IQ-7631 | 4.21E-05 | 6.98E-05 |
| TCGA-CR-7398 | 4.75E-05 | 7.41E-05 |
| TCGA-UF-A71E | 2.52E-05 | 3.36E-05 |
| TCGA-UF-A719 | 0.00041366 | 0.00045169 |
| TCGA-BA-A4IF | 0.00011111 | 0.00016391 |
| TCGA-CV-6960 | 0.00036642 | 0.00034122 |
| TCGA-CN-4725 | 0.00011874 | 0.0001443 |
| TCGA-WA-A7H4 | 1.94E-05 | 4.55E-05 |
| TCGA-CV-7103 | 0.00011892 | 5.79E-05 |
| TCGA-CV-5441 | 0.00038477 | 0.00053432 |
| TCGA-CR-7367 | 4.11E-05 | 5.78E-05 |
| TCGA-CR-5249 | 0.00011348 | 0.00011932 |
| TCGA-F7-A620 | 9.56E-05 | 0.00011413 |
| TCGA-QK-A8Z9 | 3.34E-05 | 6.86E-05 |
| TCGA-IQ-7630 | 0.00023202 | 0.00041401 |
| TCGA-CN-4738 | 0.0003935 | 0.00047392 |
| TCGA-CQ-5331 | 0.0003127 | 0.0004832 |
| TCGA-CV-7418 | 3.44E-05 | 2.58E-05 |
| TCGA-BA-A6D8 | 2.81E-05 | 3.89E-05 |
| TCGA-CV-5971 | 0.00031527 | 0.00046496 |
| TCGA-CQ-6220 | 0.0002953 | 0.00037414 |
| TCGA-QK-A8ZB | 0.0003041 | 0.0004715 |
| TCGA-CR-6481 | 8.15E-05 | 0.00014934 |
| TCGA-BB-8596 | 3.83E-05 | 7.51E-05 |
| TCGA-UF-A71D | 6.37E-05 | 8.48E-05 |
| TCGA-CV-7437 | 0.0002227 | 0.00016098 |
| TCGA-CV-A464 | 0.00023712 | 0.00039253 |
| TCGA-CV-A6JD | 2.19E-05 | 3.20E-05 |
| TCGA-CQ-7071 | 0.00012669 | 0.00028333 |
| TCGA-CV-6962 | 0.000183 | 0.00017725 |
| TCGA-CN-5366 | 7.43E-05 | 0.00010261 |
| TCGA-CN-4723 | 0.00039491 | 0.00055643 |
| TCGA-F7-A624 | 9.48E-05 | 8.81E-05 |
| TCGA-CV-A463 | 0.00012995 | 0.00013845 |
| TCGA-CV-7423 | 0.00024073 | 0.0003029 |
| TCGA-BB-4228 | 0.00023176 | 0.00029586 |
| TCGA-CV-6953 | 0.0002118 | 0.00017803 |
| TCGA-CV-7261 | 4.28E-05 | 7.80E-05 |
| TCGA-P3-A5QF | 3.05E-05 | 1.40E-05 |
| TCGA-CR-6470 | 0.0001035 | 0.00015614 |
| TCGA-CV-5979 | 0.00039174 | 0.00050108 |
| TCGA-CR-6471 | 0.00018929 | 0.00035254 |
| TCGA-CV-7430 | 0.00034264 | 0.00035533 |
| TCGA-CV-7099 | 0.00027484 | 0.00034543 |
| TCGA-CQ-6221 | 6.23E-05 | 0.00013505 |
| TCGA-QK-A6IJ | 0.00015367 | 0.00015401 |
| TCGA-CV-7406 | 0.00025364 | 0.00026363 |
| TCGA-CV-7435 | 3.85E-05 | 8.37E-05 |
| TCGA-CV-7446 | 4.32E-05 | 9.75E-05 |
| TCGA-BA-6870 | 0.00021508 | 0.00027197 |
| TCGA-MZ-A5BI | 0.00010048 | 0.00011106 |
| TCGA-CR-6478 | 8.63E-05 | 0.0001258 |
| TCGA-CN-6021 | 0.00048724 | 0.00058538 |
| TCGA-CQ-6224 | 3.66E-05 | 8.96E-05 |
| TCGA-HD-7754 | 4.55E-05 | 8.29E-05 |
| TCGA-F7-A622 | 4.81E-05 | 2.07E-05 |
| TCGA-P3-A6T2 | 3.22E-05 | 5.80E-05 |
| TCGA-CR-7383 | 1.97E-05 | 2.80E-05 |
| TCGA-CR-7379 | 0.00041143 | 0.00060165 |
| TCGA-CV-7236 | 6.27E-05 | 0.0001068 |
| TCGA-CN-4727 | 2.46E-05 | 5.45E-05 |
| TCGA-CQ-5327 | 4.30E-05 | 2.08E-05 |
| TCGA-C9-A480 | 0.00020478 | 0.00023002 |
| TCGA-CV-A6JO | 9.68E-05 | 0.00012097 |
| TCGA-CR-6492 | 0.00028193 | 0.00036127 |
| TCGA-BA-6872 | 0.00038164 | 0.00051609 |
| TCGA-BB-4223 | 5.40E-05 | 7.34E-05 |
| TCGA-CV-A6K2 | 3.11E-05 | 3.68E-05 |
| TCGA-HD-8634 | 0.00031289 | 0.00034206 |
| TCGA-CN-6994 | 0.00045 | 0.00063967 |
| TCGA-D6-A4Z9 | 0.00013997 | 0.00024594 |
| TCGA-CV-7440 | 0.00012943 | 0.00017106 |
| TCGA-CR-7376 | 0.00010382 | 0.00023306 |
| TCGA-CR-7391 | 0.00031493 | 0.00047956 |
| TCGA-T2-A6X2 | 0.0003492 | 0.00048748 |
| TCGA-BA-4074 | 0.00013849 | 0.00018265 |
| TCGA-CN-4734 | 0.00042704 | 0.00055393 |
| TCGA-CV-6441 | 3.41E-05 | 4.99E-05 |
| TCGA-CV-A6JY | 8.97E-05 | 3.08E-05 |
| TCGA-D6-6825 | 6.58E-05 | 0.00011664 |
| TCGA-CR-5247 | 5.33E-05 | 0.00010247 |
| TCGA-CV-7410 | 0.00033019 | 0.00048113 |
| TCGA-CV-7091 | 4.04E-05 | 8.15E-05 |
| TCGA-BA-5153 | 0.00064745 | 0.00082467 |
| TCGA-CV-7428 | 0.00028399 | 0.00035771 |
| TCGA-CX-7086 | 3.32E-05 | 3.20E-05 |
| TCGA-CN-5364 | 0.00037089 | 0.00048986 |
| TCGA-BA-A6DB | 0.00010787 | 0.0001655 |
| TCGA-CV-7097 | 0.00019703 | 0.00027689 |
| TCGA-CQ-5325 | 0.00038002 | 0.00054965 |
| TCGA-H7-A6C4 | 5.41E-05 | 8.07E-05 |
| TCGA-CN-4731 | 4.83E-05 | 8.41E-05 |
| TCGA-CV-7427 | 4.54E-05 | 4.13E-05 |
| TCGA-CR-6467 | 5.21E-05 | 7.45E-05 |
| TCGA-BA-5151 | 0.00037711 | 0.0005108 |
| TCGA-CV-7178 | 9.27E-05 | 0.00013232 |
| TCGA-CV-A461 | 0.00027041 | 0.0003099 |
| TCGA-BA-5559 | 0.00049138 | 0.00068496 |
| TCGA-CR-6493 | 9.37E-05 | 0.00010774 |
| TCGA-CV-A6JT | 0.00032599 | 0.0007289 |
| TCGA-UF-A7JJ | 5.19E-05 | 9.32E-05 |
| TCGA-RS-A6TO | 8.87E-05 | 0.00016879 |
| TCGA-CR-7372 | 0.00050169 | 0.00073705 |
| TCGA-D6-6827 | 3.58E-05 | 3.89E-05 |
| TCGA-QK-A6IH | 0.00035878 | 0.00055017 |
| TCGA-CN-6011 | 0.00039627 | 0.00050685 |
| TCGA-CR-6474 | 2.52E-05 | 6.79E-05 |
| TCGA-CV-6939 | 5.84E-05 | 8.33E-05 |
| TCGA-D6-8568 | 0.00040857 | 0.00056941 |
| TCGA-HD-A634 | 3.06E-05 | 1.89E-05 |
| TCGA-UF-A7JD | 6.70E-05 | 0.00010565 |
| TCGA-CV-6951 | 0.00012506 | 0.00021432 |
| TCGA-F7-8298 | 3.88E-05 | 5.81E-05 |
| TCGA-CV-6941 | 5.45E-05 | 0.00010045 |
| TCGA-H7-A76A | 2.52E-05 | 8.11E-05 |
| TCGA-D6-A6EP | 1.86E-05 | 4.07E-05 |
| TCGA-UF-A7JS | 0.00010278 | 7.22E-05 |
| TCGA-CV-A45Q | 0.00032547 | 0.00040721 |
| TCGA-QK-A6VC | 8.13E-05 | 0.00010583 |
| TCGA-BA-A6DL | 3.11E-05 | 1.73E-05 |
| TCGA-CR-7365 | 0.0004409 | 0.00062028 |
| TCGA-CV-6934 | 0.00010441 | 0.00017595 |
| TCGA-P3-A6SW | 4.69E-05 | 6.90E-05 |
| TCGA-HD-8314 | 0.00051712 | 0.00068963 |
| TCGA-CN-4742 | 3.86E-05 | 8.41E-05 |
| TCGA-IQ-A61H | 0.0003975 | 0.00052692 |
| TCGA-CX-7085 | 0.00030916 | 0.00050054 |
| TCGA-CV-7235 | 4.80E-05 | 7.08E-05 |
| TCGA-CV-7434 | 0.00017919 | 0.00027763 |
| TCGA-CR-6480 | 5.47E-05 | 3.30E-05 |
| TCGA-BB-A6UM | 7.24E-05 | 8.11E-05 |
| TCGA-C9-A47Z | 0.00037697 | 0.00042312 |
| TCGA-CR-7382 | 3.33E-05 | 5.84E-05 |
| TCGA-BB-4225 | 0.00010071 | 0.00012951 |
| TCGA-BA-A4IG | 7.76E-05 | 8.16E-05 |
| TCGA-CR-7393 | 0.00036803 | 0.00051766 |
| TCGA-T2-A6X0 | 0.00037373 | 0.00045356 |
| TCGA-F7-A623 | 0.00010975 | 0.0001587 |
| TCGA-IQ-7632 | 0.00032663 | 0.00043616 |
| TCGA-QK-A652 | 9.98E-05 | 0.00016505 |
| TCGA-WA-A7GZ | 0.0003854 | 0.00049374 |
| TCGA-CR-7388 | 0.00038612 | 0.00052552 |
| TCGA-CV-A6K1 | 3.57E-05 | 1.58E-05 |
| TCGA-CN-5363 | 0.00034821 | 0.00051609 |
| TCGA-CV-6956 | 7.87E-05 | 9.07E-05 |
| TCGA-CV-7263 | 0.00031573 | 0.00047994 |
| TCGA-CN-A49C | 6.42E-05 | 4.38E-05 |
| TCGA-IQ-A6SG | 0.00036028 | 0.0004697 |
| TCGA-UF-A7JC | 0.00040378 | 0.00047979 |
| TCGA-QK-A6II | 0.00021814 | 0.00014305 |
| TCGA-CN-A49B | 9.81E-05 | 0.00012562 |
| TCGA-IQ-A61E | 9.29E-05 | 0.00010476 |
| TCGA-CV-7101 | 0.00021873 | 0.00032473 |
| TCGA-BA-A4IH | 0.0002561 | 0.00031072 |
| TCGA-H7-8502 | 4.11E-05 | 5.59E-05 |
| TCGA-CV-7414 | 0.0001376 | 0.00020123 |
| TCGA-HD-7229 | 0.00038364 | 0.00041278 |
| TCGA-CV-6961 | 0.00046005 | 0.00066764 |
| TCGA-TN-A7HI | 0.0001115 | 0.00010679 |
| TCGA-CQ-5333 | 0.00021421 | 0.00025279 |
| TCGA-CN-4733 | 0.00026238 | 0.00037126 |
| TCGA-MT-A67F | 5.24E-05 | 5.96E-05 |
| TCGA-CR-7385 | 8.03E-05 | 9.97E-05 |
| TCGA-TN-A7HJ | 7.68E-05 | 6.86E-05 |
| TCGA-CN-4736 | 0.00036566 | 0.00056745 |
| TCGA-CN-4730 | 5.41E-05 | 8.89E-05 |
| TCGA-CV-7433 | 5.28E-05 | 9.78E-05 |
| TCGA-CN-4726 | 0.00024756 | 0.00045669 |
| TCGA-CQ-5332 | 9.25E-05 | 0.0001443 |
| TCGA-HD-8224 | 0.00050236 | 0.00071998 |
| TCGA-BB-8601 | 4.11E-05 | 5.39E-05 |
| TCGA-CN-5355 | 0.00042592 | 0.000611 |
| TCGA-QK-A6IG | 1.77E-05 | 2.07E-05 |
| TCGA-D6-6826 | 3.63E-05 | 3.76E-05 |
| TCGA-CR-7404 | 7.48E-05 | 9.75E-05 |
| TCGA-D6-A6EM | 0.00013652 | 0.00014184 |
| TCGA-CQ-A4CE | 0.00040196 | 0.00046312 |
| TCGA-CV-A460 | 0.00035751 | 0.00048465 |
| TCGA-CV-7104 | 5.64E-05 | 0.00013572 |
| TCGA-UF-A7JF | 5.57E-05 | 8.99E-05 |
| TCGA-HD-7753 | 0.00013129 | 0.00024498 |
| TCGA-F7-A61S | 4.00E-05 | 4.79E-05 |
| TCGA-CN-6017 | 0.00017841 | 0.00024817 |
| TCGA-CR-7369 | 0.00010356 | 0.00017806 |
| TCGA-P3-A6T8 | 4.39E-05 | 7.02E-05 |
| TCGA-F7-A50J | 0.00026079 | 0.00032036 |
| TCGA-CV-7100 | 0.00037095 | 0.00050214 |
| TCGA-TN-A7HL | 1.91E-05 | 5.71E-06 |
| TCGA-CV-7090 | 9.53E-05 | 0.00013189 |
| TCGA-BA-6871 | 6.94E-05 | 0.00010251 |
| TCGA-CR-7371 | 0.00044436 | 0.00064744 |
| TCGA-CV-5432 | 0.00011818 | 0.00015395 |
| TCGA-CN-5360 | 0.00049924 | 0.00066922 |
| TCGA-CQ-A4C6 | 0.00043014 | 0.000593 |
| TCGA-MT-A7BN | 5.25E-05 | 5.88E-05 |
| TCGA-CN-A641 | 8.26E-05 | 7.99E-05 |
| TCGA-CQ-A4C9 | 4.21E-05 | 5.99E-05 |
| TCGA-CQ-A4CA | 0.00026976 | 0.00038217 |
| TCGA-IQ-A61O | 4.55E-05 | 0.0001001 |
| TCGA-CQ-A4CD | 3.00E-05 | 2.73E-05 |
| TCGA-CN-A499 | 9.91E-05 | 9.25E-05 |
| TCGA-CN-6013 | 0.00035613 | 0.00048815 |
| TCGA-CN-5356 | 8.79E-05 | 0.00014738 |
| TCGA-CV-7242 | 0.0003172 | 0.00035052 |
| TCGA-CN-5369 | 5.93E-05 | 0.00014965 |
| TCGA-CN-6988 | 5.05E-05 | 5.96E-05 |
| TCGA-D6-A6EN | 2.48E-05 | 1.23E-05 |
| TCGA-CQ-A4C7 | 6.35E-05 | 9.37E-05 |
| TCGA-CV-5435 | 0.00055553 | 0.00078031 |
| TCGA-CR-7370 | 0.00055451 | 0.00076021 |
| TCGA-CN-4722 | 0.00029915 | 0.00041185 |
| TCGA-CN-6023 | 2.40E-05 | 1.65E-05 |
| TCGA-CQ-A4CI | 0.00031929 | 0.00046237 |
| TCGA-CV-7438 | 0.00023247 | 0.00034676 |
| TCGA-BB-4224 | 0.00020153 | 0.0001809 |
| TCGA-CN-6997 | 0.00053802 | 0.00073639 |
| TCGA-CV-6937 | 0.00033337 | 0.00043794 |
| TCGA-CQ-5326 | 7.97E-05 | 0.00017131 |
| TCGA-BA-A6DG | 0.00022082 | 0.0003325 |
| TCGA-UF-A7JT | 5.94E-05 | 9.87E-05 |
| TCGA-CN-A6V6 | 0.00026384 | 0.00033524 |
| TCGA-CR-6473 | 3.13E-05 | 2.20E-05 |
| TCGA-BA-7269 | 3.20E-05 | 4.93E-05 |
| TCGA-CV-7177 | 0.00055101 | 0.0005423 |
| TCGA-BB-A6UO | 8.55E-05 | 0.00010262 |
| TCGA-CV-A468 | 0.00017214 | 0.00022507 |
| TCGA-CQ-5324 | 6.44E-05 | 8.91E-05 |
| TCGA-CQ-A4CH | 3.44E-05 | 5.89E-05 |
| TCGA-CN-6020 | 0.0004356 | 0.00064396 |
| TCGA-CN-A63V | 4.78E-05 | 7.15E-05 |
| TCGA-CV-7415 | 0.00043758 | 0.00058438 |
| TCGA-CV-6003 | 6.83E-05 | 5.15E-05 |
| TCGA-CN-6016 | 7.74E-05 | 0.00017815 |
| TCGA-CV-5966 | 3.80E-05 | 4.61E-05 |
| TCGA-BA-4076 | 0.0002272 | 0.00027083 |
| TCGA-CN-4735 | 0.00036762 | 0.00049052 |
| TCGA-CV-6955 | 0.00031207 | 4.15E-05 |
| TCGA-CR-6491 | 0.00028733 | 0.00044502 |
| TCGA-BA-5558 | 0.00035778 | 0.00046804 |
| TCGA-CN-4728 | 3.92E-05 | 7.89E-05 |
| TCGA-BB-A5HZ | 0.00010293 | 0.00016294 |
| TCGA-UP-A6WW | 8.65E-05 | 0.00014273 |
| TCGA-CV-7407 | 0.00026774 | 0.00029357 |
| TCGA-CV-6936 | 0.00021311 | 0.00023578 |
| TCGA-CV-7416 | 7.12E-05 | 9.68E-05 |
| TCGA-CN-5361 | 0.00032968 | 0.00052635 |
| TCGA-CN-A49A | 2.91E-05 | 4.68E-05 |
| TCGA-CN-6998 | 0.00039966 | 0.000524 |
| TCGA-MZ-A6I9 | 5.90E-05 | 5.40E-05 |
| TCGA-F7-A50I | 0.00022711 | 0.00032139 |
| TCGA-T2-A6WX | 8.71E-05 | 0.00016785 |
| TCGA-CQ-6229 | 0.00041109 | 0.00055266 |
| TCGA-CN-A63U | 5.24E-05 | 5.91E-05 |
| TCGA-CV-6940 | 0.00045549 | 0.0006572 |
| TCGA-H7-8501 | 3.27E-05 | 3.29E-05 |
| TCGA-CV-7255 | 0.00038858 | 0.00049067 |
| TCGA-CR-5243 | 0.00063899 | 0.00088185 |
| TCGA-P3-A6T5 | 7.33E-05 | 0.00013026 |
| TCGA-CV-6952 | 0.00031853 | 0.00040254 |
| TCGA-D6-A6ES | 0.00083337 | 0.00051874 |
| TCGA-UF-A7J9 | 2.82E-05 | 6.59E-05 |
| TCGA-CQ-6225 | 0.00019999 | 0.00017022 |
| TCGA-MT-A51X | 0.00038441 | 0.00055909 |
| TCGA-BA-5152 | 4.65E-05 | 7.23E-05 |
| TCGA-CQ-7065 | 0.00028991 | 0.00032292 |
| TCGA-CN-4741 | 0.0001841 | 0.00030325 |
| TCGA-CR-7380 | 9.48E-05 | 0.00014902 |
| TCGA-F7-A61W | 1.91E-05 | 1.51E-05 |
| TCGA-CV-5439 | 0.00027189 | 0.00027443 |
| TCGA-HD-A4C1 | 9.95E-05 | 0.00012382 |
| TCGA-D6-6517 | 0.00040478 | 0.00049378 |
| TCGA-CQ-6227 | 3.92E-05 | 5.18E-05 |
| TCGA-CV-7089 | 0.00011098 | 0.00010358 |
| TCGA-QK-AA3K | 1.50E-05 | 3.16E-05 |
| TCGA-CN-5367 | 0.00016277 | 0.00024583 |
| TCGA-CN-5365 | 0.00019375 | 0.00021616 |
| TCGA-CV-7095 | 0.00017801 | 0.00027092 |
| TCGA-QK-A8ZA | 3.93E-05 | 4.02E-05 |
| TCGA-CV-A6JE | 0.00015927 | 0.00019557 |
| TCGA-CQ-7063 | 0.00014278 | 0.00016079 |
| TCGA-BB-4227 | 6.97E-05 | 8.70E-05 |
| TCGA-CV-6433 | 3.28E-05 | 1.22E-05 |
| TCGA-CN-5373 | 0.00044435 | 0.00073594 |
| TCGA-CV-7568 | 0.00035192 | 0.00054453 |
| TCGA-CN-6989 | 3.80E-05 | 6.11E-05 |
| TCGA-CV-7424 | 5.04E-05 | 8.41E-05 |
| TCGA-BA-4078 | 0.00029952 | 0.00044197 |
| TCGA-CN-4739 | 8.44E-05 | 0.00012733 |
| TCGA-CV-5430 | 8.17E-05 | 9.38E-05 |
| TCGA-P3-A6SX | 6.51E-05 | 7.64E-05 |
| TCGA-CV-7252 | 0.00041265 | 0.00043339 |
| TCGA-D6-A6EQ | 0.00014236 | 0.00018102 |
| TCGA-CN-6995 | 8.86E-05 | 0.00011998 |
| TCGA-KU-A6H7 | 8.23E-05 | 6.13E-05 |
| TCGA-D6-A6EO | 5.27E-05 | 7.62E-05 |
| TCGA-KU-A6H8 | 0.00020843 | 0.0002348 |
| TCGA-CN-6010 | 0.00042116 | 0.00053342 |
| TCGA-BA-A8YP | 4.03E-05 | 6.19E-05 |
| TCGA-D6-A4ZB | 0.00011143 | 0.00013486 |
| TCGA-CV-7422 | 0.00035828 | 0.00047624 |
| TCGA-CX-A4AQ | 0.0001761 | 0.00022986 |
| TCGA-CQ-5330 | 5.01E-05 | 7.90E-05 |
| TCGA-P3-A6T3 | 6.68E-05 | 3.48E-05 |
| TCGA-BB-A5HY | 4.17E-05 | 5.38E-05 |
| TCGA-CV-A45R | 0.0002487 | 0.00041121 |
| TCGA-P3-A6T4 | 0.00036624 | 0.0004443 |
| TCGA-MT-A51W | 0.00036385 | 0.00044158 |
| TCGA-CQ-7068 | 8.45E-05 | 0.00010938 |
| TCGA-4P-AA8J | 4.95E-05 | 6.04E-05 |
| TCGA-CV-A45P | 0.00017749 | 0.00018962 |
| TCGA-CV-7429 | 2.76E-05 | 3.88E-05 |
| TCGA-HD-A633 | 6.59E-05 | 7.99E-05 |
| TCGA-DQ-5631 | 0.00067637 | 0.00091493 |
| TCGA-CV-5436 | 0.00017203 | 0.00024649 |
| TCGA-DQ-5630 | 0.00022737 | 0.00034649 |
| TCGA-CR-6482 | 6.07E-05 | 8.29E-05 |
| TCGA-P3-A6T6 | 2.36E-05 | 2.66E-05 |
| TCGA-DQ-7592 | 0.00052349 | 0.0006922 |
| TCGA-BA-A4II | 0.00037442 | 0.00048505 |
| TCGA-HL-7533 | 7.14E-05 | 0.00011876 |
| TCGA-D6-6516 | 0.0003926 | 0.00045629 |
| TCGA-CV-A45V | 0.00036123 | 0.00049126 |
| TCGA-H7-7774 | 0.00028791 | 0.00024829 |
| TCGA-CQ-A4CG | 6.49E-05 | 0.00011507 |
| TCGA-CV-5970 | 0.0005238 | 0.00075376 |
| TCGA-BB-4217 | 6.90E-05 | 6.29E-05 |
| TCGA-CR-5248 | 5.17E-05 | 5.61E-05 |
| TCGA-CR-7394 | 5.72E-05 | 0.00011707 |
| TCGA-CV-7421 | 0.00032225 | 0.00044929 |
| TCGA-CN-4729 | 0.00024247 | 0.00029107 |
| TCGA-T2-A6WZ | 9.09E-05 | 0.00011448 |
| TCGA-CV-6436 | 0.00021985 | 0.00022432 |
| TCGA-UF-A7JA | 3.62E-05 | 5.75E-05 |
| TCGA-CQ-A4CB | 4.53E-05 | 6.12E-05 |
| TCGA-CX-7219 | 3.67E-05 | 6.05E-05 |
| TCGA-IQ-A61G | 5.41E-05 | 5.77E-05 |
| TCGA-CV-5434 | 0.00031488 | 0.00045943 |
| TCGA-RS-A6TP | 3.40E-05 | 1.73E-05 |
| TCGA-CV-5978 | 3.62E-05 | 5.74E-05 |
| TCGA-CV-5977 | 0.00027553 | 0.00032683 |
| TCGA-CV-A6JU | 1.48E-05 | 1.66E-05 |
| TCGA-CV-7102 | 8.13E-05 | 9.88E-05 |
| TCGA-CR-7368 | 0.00023327 | 0.0002971 |
| TCGA-D6-A74Q | 0.00021847 | 0.00031813 |
| TCGA-D6-6824 | 0.00037729 | 0.00057291 |
| TCGA-CV-5444 | 4.32E-05 | 7.26E-05 |
| TCGA-CN-A6V7 | 6.01E-05 | 5.86E-05 |
| TCGA-CV-A45Z | 0.00020274 | 0.00024076 |
| TCGA-BA-A6DE | 0.00034777 | 0.00040127 |
| TCGA-D6-8569 | 4.77E-05 | 9.33E-05 |
| TCGA-CR-7401 | 0.0001073 | 0.00023056 |
| TCGA-CV-7247 | 7.04E-05 | 0.00014535 |
| TCGA-CQ-7072 | 2.99E-05 | 6.48E-05 |
| TCGA-CV-A45T | 6.18E-05 | 5.13E-05 |
| TCGA-BA-4075 | 9.69E-06 | 2.18E-05 |
| TCGA-DQ-5624 | 0.00041072 | 0.00055169 |
| TCGA-BA-A6DD | 0.00022015 | 0.00029341 |
| TCGA-KU-A66S | 2.97E-05 | 3.76E-05 |
| TCGA-CV-7254 | 0.00021819 | 0.00031507 |
| TCGA-CV-5976 | 5.38E-05 | 7.51E-05 |
| TCGA-CR-7374 | 5.95E-05 | 7.38E-05 |
| TCGA-CR-5250 | 9.64E-05 | 0.00015652 |
| TCGA-CV-A6JZ | 4.69E-05 | 0.00011723 |
| TCGA-CV-6954 | 4.32E-05 | 6.04E-05 |
| TCGA-P3-A5QE | 4.15E-05 | 4.78E-05 |
| TCGA-QK-A8Z8 | 0.00038476 | 0.00042823 |
| TCGA-CV-A465 | 0.00019511 | 0.0001478 |
| TCGA-DQ-5629 | 0.00039722 | 0.0005538 |
| TCGA-BA-A6DJ | 0.00021626 | 0.00027038 |
| TCGA-CR-7395 | 0.00040701 | 0.000538 |
| TCGA-CQ-6223 | 0.00015858 | 0.00014901 |
| TCGA-F7-7848 | 2.76E-05 | 5.18E-05 |
| TCGA-CV-A6JM | 0.0002818 | 0.00022468 |
| TCGA-CN-5359 | 2.85E-05 | 6.17E-05 |
| TCGA-P3-A5QA | 6.05E-05 | 8.75E-05 |
| TCGA-CV-6948 | 3.23E-05 | 4.68E-05 |
| TCGA-QK-A64Z | 9.40E-05 | 0.00013875 |
| TCGA-CQ-6228 | 0.00028118 | 0.00036851 |
| TCGA-CN-6996 | 0.00015487 | 0.00025412 |
| TCGA-CV-5431 | 7.67E-05 | 0.00013335 |
| TCGA-CV-A45O | 0.00083953 | 0.00102537 |
| TCGA-T3-A92N | 6.09E-05 | 8.33E-05 |
| TCGA-CR-7399 | 0.00029311 | 0.00033195 |
| TCGA-CQ-5334 | 6.53E-05 | 0.00010357 |
| TCGA-CR-7402 | 5.95E-05 | 6.28E-05 |
| TCGA-CR-7389 | 0.00045255 | 0.00063669 |
| TCGA-BA-6868 | 0.00039301 | 0.00054853 |
| TCGA-CR-6488 | 7.51E-05 | 0.00012803 |
